# Analyzing miRNA co-expression networks to explore TF-miRNA regulation

**DOI:** 10.1186/1471-2105-10-163

**Published:** 2009-05-28

**Authors:** Sanghamitra Bandyopadhyay, Malay Bhattacharyya

**Affiliations:** 1 Machine Intelligence Unit, Indian Statistical Institute, 203 BT Road, Kolkata, 700108, India

## Abstract

**Background:**

Current microRNA (miRNA) research in progress has engendered rapid accumulation of expression data evolving from microarray experiments. Such experiments are generally performed over different tissues belonging to a specific species of metazoan. For disease diagnosis, microarray probes are also prepared with tissues taken from similar organs of different candidates of an organism. Expression data of miRNAs are frequently mapped to co-expression networks to study the functions of miRNAs, their regulation on genes and to explore the complex regulatory network that might exist between Transcription Factors (TFs), genes and miRNAs. These directions of research relating miRNAs are still not fully explored, and therefore, construction of reliable and compatible methods for mining miRNA co-expression networks has become an emerging area. This paper introduces a novel method for mining the miRNA co-expression networks in order to obtain co-expressed miRNAs under the hypothesis that these might be regulated by common TFs.

**Results:**

Three co-expression networks, configured from one patient-specific, one tissue-specific and a stem cell-based miRNA expression data, are studied for analyzing the proposed methodology. A novel compactness measure is introduced. The results establish the statistical significance of the sets of miRNAs evolved and the efficacy of the self-pruning phase employed by the proposed method. All these datasets yield similar network patterns and produce coherent groups of miRNAs. The existence of common TFs, regulating these groups of miRNAs, is empirically tested. The results found are very promising. A novel visual validation method is also proposed that reflects the homogeneity as well as statistical properties of the grouped miRNAs. This visual validation method provides a promising and statistically significant graphical tool for expression analysis.

**Conclusion:**

A heuristic mining methodology that resembles a clustering motivation is proposed in this paper. However, there remains a basic difference between the mining method and a clustering approach. The heuristic approach can produce priority modules (*PM*) from an miRNA co-expression network, by employing a self-pruning phase, which are analyzed for statistical and biological significance. The mining algorithm minimizes the space/time complexity of the analysis, and also handles noise in the data. In addition, the mining method reveals promising results in the unsupervised analysis of TF-miRNA regulation.

## Background

Throughout the last decade, much research was devoted to unearth the functionality of microRNAs (miRNAs), which are small (21–23 nt), non-coding RNAs regulating mRNA stability and translation through the action of the RNA-induced silencing complex (RISC) [1-3]. Earlier investigations [2,4] have discovered that miRNAs regulate a variety of key biological functions that includes insulin secretion, apoptosis, cell proliferation and differentiation, etc. More importantly, recent beliefs hypothesize that miRNAs are indirectly responsible, due to disorders in functionality, for a number of diseases as they can dysregulate post-transcriptional gene expression [5]. Emerging evidences suggest that miRNAs regulate brain development, dendritic spine morphology, and neurite outgrowth, i.e., certain processes that are hypothesized to be associated with schizophrenia neuropathology. Moreover, they also have influencing activities in regulating the diseases like Tourette's syndrome, Fragile × syndrome [2], several varieties of cancers [4] and many others [5].

Microarray profiling is a high-throughput experimentation that can be used to study the expressibility/repressibility measure of thousands of genes in parallel [6,7]. In the recent past, microarray data has been studied extensively for gene expression analysis leading to many methodological works. But the field of analyzing miRNA microarrays is not well-explored. The expression profiles of miRNAs derived from microarray experiments are most of the times tissue-specific in nature. In addition, miRNAs are sometimes taken for expression profiling from common tissues (by locality) of different patients for the purpose of disease diagnosis. Not surprisingly, due to the short length of miRNAs, the purity, variance and dimension of the microarray datasets of miRNAs are smaller than those of the genes. Thus, developing efficient methods that could shed light into the underlying biological activity of miRNAs is imperative, without depending on the methods developed for gene expression data [7-9].

A natural approach in microarray study is mapping the simultaneous overexpression/underexpression of miRNA pairs into a co-expression network. These co-expression networks are analyzed to study the functional enrichment and regulatory activities of miRNAs [10,11]. However, the most important (and ignored) target remains in preparing the blueprint of the complex regulatory network that hypothetically exists between transcription factors (TFs), genes and miRNAs. Some of the earlier studies advocated that the miRNAs targeting the same gene together with a TF might be regulated by the same TF [12]. By exercising on the established knowledge in TRANSFAC database and microRNA registry, an earlier study was done on TF and miRNA regulation relating to prostate cancer cells [13]. A recent study pursues the same hypothesis adding that there are TF-miRNA pairs that participate in a complex recurring network and exert regulatory effects on each other [3]. But, these previous analyzes either follow supervised learning based on the established results available in the databases like TargetScan [14] and PicTar [15] or lack exhaustive empirical study. There exists an impressive number of works on clustering miRNA co-expression networks with various motivations like identification of the set of miRNAs derived from common primary transcripts [10], co-expression analysis between neighboring miRNAs [11], study of diseases [4], co-expression analysis of miRNA with mRNA [16], etc. Again, these approaches do not target the construction of TF-miRNA regulatory networks. Moreover, they employ clustering tools commonly used for gene expression analysis though, as mentioned earlier, the scalability and the other characteristics of miRNA expression data are somewhat different.

This paper introduces a novel unsupervised mining method that can heuristically self-prune a co-expression network constructed from miRNA profiled microarray data. The iterative mining methodology produces a set of priority modules (*PM*s) from the dataset. The statistical (and hypothetically the biological) significance of the *PM*s decreases as they are generated by stepwise reduction. The results show that the transcription factor binding sites (TFBSs) of the grouped miRNAs in the 5' untranscribed region (UR) have large common portions establishing the existence of commonly regulating TFs. In a recent work having similar goal, clustering of miRNAs was done based on their commonalties in loci [3]. Evidently, their defined putative upstream region (<10 kb) will contain a large number of common TFs for the clustered miRNAs. This was a kind of supervised approach, and from this viewpoint the mining process discussed here is a novel one of its kind. A schizophrenia patient-specific, a tissue-specific and a stem cell-based microarray dataset are comprehensively analyzed. The studies show that these datasets are useful to explore common TFs which might regulate a module of miRNAs. Such TF-miRNA regulation information might in turn accelerate the reconstruction of TF-miRNA regulatory networks.

A network (in general, a weighted undirected network) is often defined by the triplet (*N*, *A*, *W*), where *N *denotes a finite set of nodes {*n*_1_, *n*_2_,..., *n*_|*N*|_} (cardinality of the set *N *is represented as |*N*|),  denotes a set of edges between the node pairs, and *W*: *A *→ [0, ∞) is a weight function associated with the edges. Here, a network,  = (*N*, *A*, *W*), is referred to as an miRNA co-expression network if the node set (*N*) corresponds to a set of miRNAs and *W *: *A *→ [0, 1] denotes a co-expression function mapped from each miRNA pair in *A*.

In general, miRNA co-expression networks can be thought of as fuzzy complete graphs [17] by excluding the arcs having a co-expression value of zero. This transformation occurs by the mapping of miRNAs to the vertices and co-expression values to the fuzzy membership values. Thus a module identified in a fuzzy complete graph will evidently denote a set of miRNAs by such transformation. A recent study proposes an *O*(*n*^2 ^log *n*) algorithm for identifying the largest dense N-vertexlet (a set of vertices of cardinality *N*, ), in a fuzzy scale-free graph [17]. The miRNA co-expression networks, being of this nature, could be mined step by step using a similar approach. For describing the proposed mining process that integrates this earlier work [17], the following theoretical details are given.

**Definition 1 (Fuzzy Complete Graph) ***A fuzzy complete graph (FCG),  = (V, , Ω), is defined as a graph in which V denotes the set of vertices,  denotes the set of fuzzy relations (v_i_, v_j_) (v_i _≠ v_j_, ∀v_*i*_, v_*j *_∈ V) and Ω is a fuzzy membership function defined over the set  such that Ω*:  → (0, 1].

**Definition 2 (Association Density of a vertex) ***Given an FCG,  = (V, , Ω), the association density,  of a vertex v_*i *_of  is defined, with respect to a set of vertices  (v_*i *_∉ ), as the ratio of the sum of the fuzzy edge memberships between v_*i *_and each of the vertices belonging to  and N. Thus, the association density of a vertex v_*i *_with respect to  is computed as*,

(1)

In Eqn. (1),  denotes the fuzzy membership value of the edge (*v*_*i*_, *v*_*j*_). This density definition computes the degree of participation of a single vertex with respect to a set of vertices. By putting the constraint of a lower bound to this density factor for every vertex within a group of vertices, the association density of an N-vertexlet is now defined as follows.

**Definition 3 (Association Density of an N-vertexlet) ***The association density of an N-vertexlet  is defined to be the minimum of the association density of every vertex belonging to the N-vertexlet with respect to the remaining (N-1)-vertexlet. So, the association density of an N-vertexlet  is given by,*

(2)

Suppose, an arbitrary association density value *δ *is given. If the association density of an N-vertexlet, , equals or exceeds *δ*, then  is called a dense N-vertexlet with respect to *δ *and is denoted as (*δ*). The proposed method derives a set of modules comprising a set of vertices (corresponding to miRNAs here) which are equivalent to such dense N-vertexlets. Thus, the proposed method mines an FCG for identifying the dense N-vertexlets which are equivalent to finding modules in an miRNA co-expression network. Let an arbitrary FCG induced by the node set *N'*, in an FCG  = (*V*, , Ω), be denoted as , where  and Ω_*N' *_are the edge set and the fuzzy membership function induced by the node set *N' *in  and Ω respectively. Then, a set of *PM*s in this FCG is defined as follows.

**Definition 4 (Priority Modules) ***Given an FCG,  = (V, , Ω), mapped from an miRNA co-expression network, a set of k priority modules (PMs) {} () is defined such that*,

1. ,

2. ,

3. .

The basic goal of this work is determining a significant set of *PM*s from the miRNA microarray profiled data for the unsupervised analysis of the TF-miRNA regulation.

## Results and discussion

**Figure 1:**
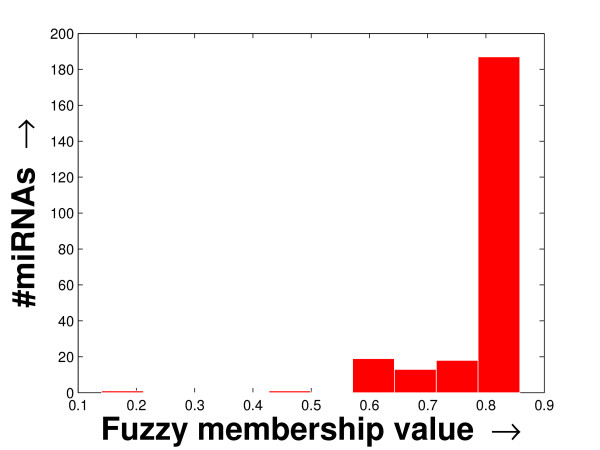
**Histogram of the column-specific FCG constructed from the schizophrenia dataset**. The histogram of the column-specific fuzzy membership values as derived in the FCG constructed from the schizophrenia dataset. The average fuzzy membership values of all the miRNAs with respect to the other miRNAs are computed. Then the histogram is prepared by plotting the number of miRNAs against the average fuzzy membership value computed.

**Figure 2:**
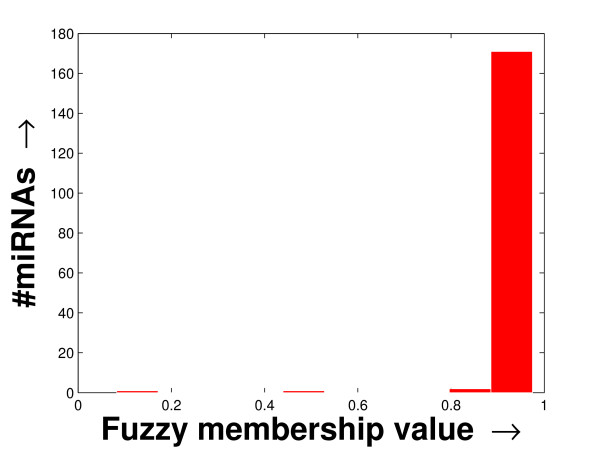
**Histogram of the column-specific FCG constructed from the tissue-specific dataset**. The histogram of the column-specific fuzzy membership values as derived in the FCG constructed from the tissue-specific dataset. The average fuzzy membership values of all the miRNAs with respect to the other miRNAs are computed. Then the histogram is prepared by plotting the number of miRNAs against the average fuzzy membership value computed.

**Figure 3:**
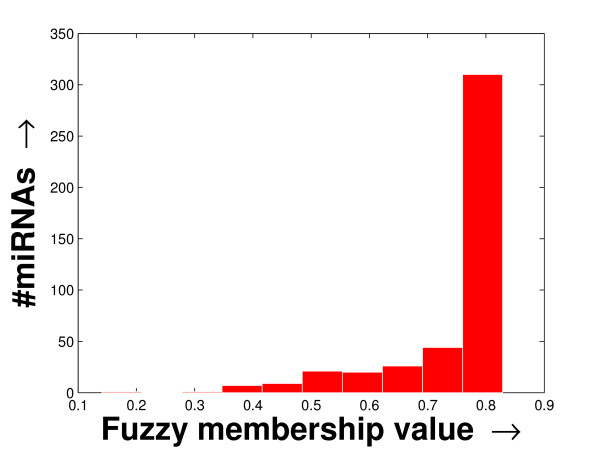
**Histogram of the column-specific FCG constructed from the stem cell dataset**. The histogram of the column-specific fuzzy membership values as derived in the FCG constructed from the stem cell dataset. The average fuzzy membership values of all the miRNAs with respect to the other miRNAs are computed. Then the histogram is prepared by plotting the number of miRNAs against the average fuzzy membership value computed.

The experimentation has been carried out on three separate FCGs derived from a schizophrenia patient-specific microarray dataset [2], one tissue-specific microarray dataset [11] and another stem cell dataset [18] (details given in Additional file 1 section 1). Due to the noisy nature of microarray experiments, often microarray expression profiling contains missing values. Here, we use the Bayesian principal component analysis (BPCA), which is a good one according to a recent study [19], for the imputation of missing values present only in the tissue-specific dataset. Then, the FCGs have been constructed by computing the fuzzy membership values (Eqn. (6)) between every miRNA pair. These FCGs can be equivalently considered as co-expression networks to be explored. The histogram of the average fuzzy membership values of the miRNAs with respect to others (details in Additional file 1 section 2.2) computed in the case of all three FCGs are shown in Figures 1, 2 and 3. In all these histograms, the distribution of the number of miRNAs follow a long tail with the decrease in fuzzy membership values. They also indicate that only a small fragment of the miRNAs is statistically significant, within which the mean of the fuzzy membership shows higher value and nominal variance.

Again, the fuzzy membership values of the miRNAs, over all the tissues/patients, are computed for these datasets. These fuzzy membership values of the miRNAs for all the experiments, in the form of a histogram (shown in Additional file 1 section 2.2), reflect that a large number of miRNA pairs are highly co-expressed. The distributions of the miRNA sizes reflected in these histograms against the fuzzy membership values help to select the lower density threshold (*δ*_*lower*_) and the density decay constant (*ξ*) employed by the proposed method. By studying the histograms, we selected *δ*_*lower *_= 0.95 and *ξ *= 0.005 (for smooth tail) for the schizophrenia dataset, *δ*_*lower *_= 0.99 and *ξ *= 0.001 (for sharp tail) for the tissue-specific dataset, and *δ*_*lower *_= 0.93 and *ξ *= 0.003 (for smooth tail) for the stem cell dataset.

After tuning the controlling parameters of the algorithm, we now mine these FCGs (representing miRNA co-expression networks) using the self-pruning method described in the algorithm provided in Table 1. The post-processing routine is iterated for 500 times. The module sizes found by the algorithm from all the three datasets are evenly distributed without containing tiny miRNA modules (single miRNA or an miRNA pair). The degraded density values derived at each time step along with the sizes of the *PM*s found from all the datasets are shown in Tables 2, 3, 4. It may be noted that the consecutive *PM*s generated by the algorithm will be in a decreasing order of statistical significance. Thus, the intra-cluster homogeneity [20] should be higher, or in effect the squared error (*SE*) should be smaller, for the *PM*s generated earlier. The *SE *value of any arbitrary *PM*, *C*, is computed as,

**Table 1:** An unsupervised algorithm for mining FCGs mapped from miRNA co-expression networks

**Input: **An FCG = (*V*, , Ω), a lower density threshold = *δ*_*lower *_and a density decay constant *ξ*.
**Output: **A set of *k *number of *PM*s {}.
**Formal steps:**
1: Set *t *← 0
2: Set *δ*_*t *_← 1
3: **while ***δ*_*t *_≥ *δ*_*lower *_**do**
4: Find the largest *PM*, , from with respect to the association density *δ*_*t*_
5: **if ** ≠ *ɸ ***then**
6: *V *← *V *-
7:
8: **end if**
9: *t *← *t *+ 1
10: *δ*_*t *_← *δ*_*t*_(1 - *ξ*)
11: **end while**
12: *k *← *t*

**Table 2:** The *PM*s obtained by applying the mining method over the schizophrenia dataset

*t*	*δ*_*t*_	Module size	*SE*	Σ*SE*	*SI*_*C*/*V*_
0	1	-	-	-	-
...	...	-	-	-	-
3	0.9850	13	0.18	70.32	0.9966
4	0.9802	8	0.23	69.79	0.9953
5	0.9752	26	0.49	72.71	0.9913
6	0.9704	15	0.69	71.89	0.9857
7	0.9655	4	0.85	70.25	0.9758
8	0.9607	14	1.25	72.51	0.9729
9	0.9559	25	1.22	72.86	0.9786
10	0.9511	6	1.37	64.76	0.9904

The *PM*s obtained by applying the mining method over the schizophrenia dataset. The values of *δ*_*t *_and *SI*_*C/V *_are rounded off upto 4 decimal places.

**Table 3:** The *PM*s obtained by applying the mining method over the tissue-specific dataset

*t*	*δ*_*t*_	Module size	*SE*	Σ*SE*	*SI*_*C/V*_
0	1	14	3.68	1.31E6	1.0000
1	0.9990	20	26.72	1.36E6	1.0000
2	0.9980	29	139.19	1.44E6	0.9998
3	0.9970	16	489.49	1.32E6	0.9992
4	0.9950	17	1.42E3	1.33E6	0.9978
5	0.9940	3	1.47E3	1.23E6	0.9965
6	0.9920	9	3.75E3	1.27E6	0.9935
7	0.9900	10	5.87E3	1.28E6	0.9899

The *PM*s obtained by applying the mining method over the tissue-specific dataset. The values of *δ*_*t *_and *SI*_*C/V *_are rounded off upto 4 decimal places.

**Table 4:** The *PM*s obtained by applying the mining method over the stem cell dataset

*t*	*δ*_*t*_	Module size	*SE*	Σ*SE*	*SI*_*C/V*_
0	1	-	-	-	-
1	0.9970	4	4.23E5	7.26E9	0.9999
2	0.9940	20	2.0E6	7.4E9	0.9996
3	0.9910	16	4.37E6	7.38E9	0.9991
4	0.9881	10	8.53E6	7.33E9	0.9982
5	0.9851	14	1.13E7	7.37E9	0.9977
6	0.9821	17	1.73E7	7.41E9	0.9965
7	0.9733	24	4.04E7	7.52E9	0.9920
8	0.9646	8	6.31E7	7.38E9	0.9853
9	0.9617	14	6.78E7	7.43E9	0.9863
10	0.9588	15	8.0E7	7.43E9	0.9848
11	0.9531	20	1.17E8	7.56E9	0.9757
12	0.9417	12	1.68E8	7.54E9	0.9609
13	0.9304	7	2.53E8	7.58E9	0.9313

The *PM*s obtained by applying the mining method over the stem cell dataset. The values of *δ*_*t *_and *SI*_*C/V *_are rounded off upto 4 decimal places.

(3)

Obviously, the value of *SE *ranges within [0, ∞). Higher the *SE *value, lower is the compactness of the *PM*. Again, the squared error of a solution with k modules (Σ*SE*) is computed as,

(4)

**Figure 4:**
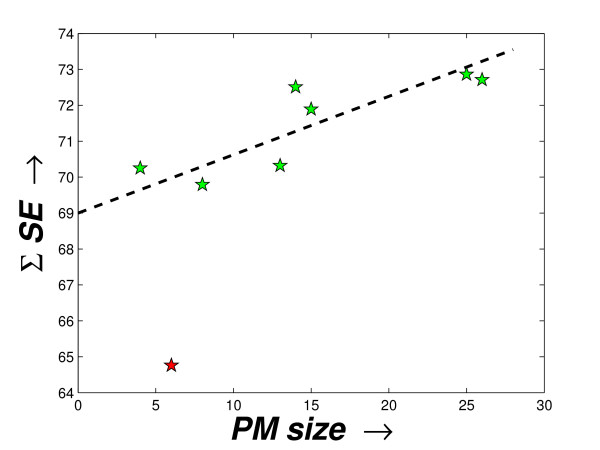
**Sizes of the *PM*s vs. the Σ*SE *score of the modules produced for the schizophrenia dataset**. The plot shows the sizes of the *PM*s vs. the Σ*SE *scores computed from these *PM*s as derived by the proposed method from the schizophrenia dataset.

**Figure 5:**
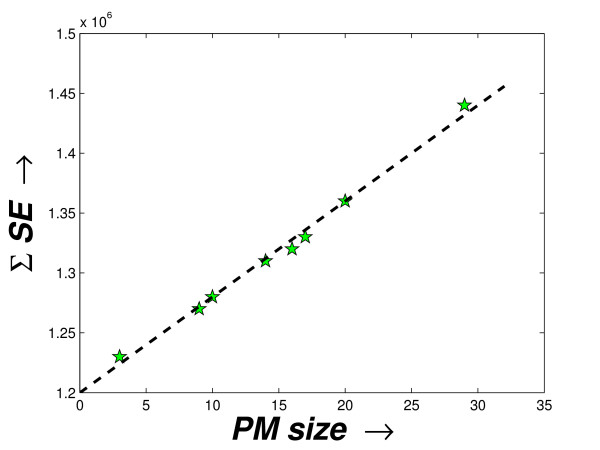
**Sizes of the PMs vs. the Σ*SE *score of the modules produced for the tissue-specific dataset**. The plot shows the sizes of the *PM*s vs. the Σ*SE *scores computed from these *PM*s as derived by the proposed method from the tissue-specific dataset.

**Figure 6:**
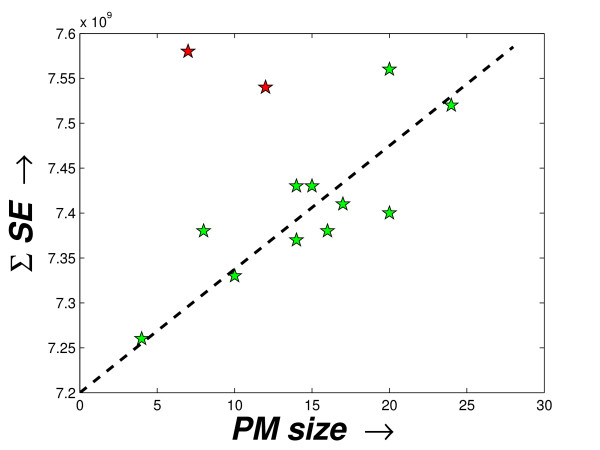
**Sizes of the PMs vs. the Σ*SE *score of the modules produced for the stem cell dataset**. The plot shows the sizes of the *PM*s vs. the Σ*SE *scores computed from these *PM*s as derived by the proposed method from the stem cell dataset.

Here, the computation of Σ*SE *is done by assuming that each *PM *produces a separate 2-cluster solution. The first one is the *PM*, itself and the second cluster contains the background set of miRNAs. To show the decreasing compactness in the *PM*s, these two measures are used and the values computed for the three datasets are shown in the fourth and fifth columns in the Tables 2, 3, 4, respectively. As expected, the *SE *values of the *PM*s generally increase in the order of their derivation. Only for the schizophrenia dataset, for *δ*_*t *_= 0.9559, a decrease in *SE *may be noted. The value Σ*SE *derived for the priority modules with respect to the sizes of the *PM*s is shown in Figures 4, 5 and 6. Notably, there is a direct dependence of the Σ*SE *values on the *PM *sizes (a single exception (outlier) observed for the schizophrenia dataset and two exceptions (outliers) for the stem cell dataset). With the reduction in the size of the *PM*s, a larger compact set is introduced in the background module. Although this causes a decrease in the *SE *value of the *PM*, but the *SE *value of the background module increases. However, this increase must be relatively smaller than the decrease in the *SE *of the *PM *as the Σ*SE *value reduces with size (observed from Figures 4, 5 and 6).

Another important clustering index, the Silhouette Index [7,21], is measured to verify the inter-cluster dissimilarity between the *PM*s found. Often, the Silhouette Index (*SI*_*C*/*V*_) is defined for a single cluster *C *with respect to a background set *V *[17]. Using this measure, the *SI*_*C*/*V *_values have been computed (details in Additional file 1 section 2.3) for the *PM*s derived from all the datasets and are given in the last columns of the Tables 2, 3, 4. The value of *SI*_*C*/*V *_ranges within [-1,+1], with higher values indicating better mined modules. As expected, the values of *SI*_*C*/*V *_of the *PM*s generally decrease in the order of their derivation. Some exceptions in this trend may be noted (from Table 2 and Table 4) for the schizophrenia and stem cell dataset for the last few *PM*s, as was also seen in the case of *SE *values. This might be due to the selection of lower density threshold (*δ*_*lower*_) which is required to be tuned more tightly.

The sizes of the miRNA groups found are validated following a method of deriving the upper bound of a clique of a graph (see Additional file 1 section 2.4) introduced in [22]. The upper bound is found to be 119 by setting *δ *= 0.95, 121 by setting *δ *= 0.99, and 187 by setting *δ *= 0.93 for the schizophrenia dataset, tissue-specific and stem cell datasets, respectively. These are the expected sizes of the most compact miRNA modules present in the networks. From the pruning method we have used, the sizes of the significant set of miRNAs are found as 111 (~46%), 118 (~67%) and 181 (~41%). These are significantly similar to the upper bounds derived theoretically, and thus important.

The motivation of the current work may bias the importance of the mining method by suggesting that it is suitable only for the miRNA expression data or scalable up to their standard size (as miRNA expression datasets have lower dimensions than the gene expression datasets). But, this is not the case. The procedure is equally good for a gene expression dataset. The miRNA expression datasets are studied here to motivate our hypothesis on TF-miRNA regulation. However, for verifying the effectiveness of the proposed method a gene expression dataset was considered. This dataset consists of expression values of 6167 genes over 52 time points (details in Additional file 1 section 2.5). The results show that the proposed method is well applicable to this larger dataset indicating its scalability. Moreover, the discussion on the algorithmic complexity (see Additional file 1 section 2.5) highlight that it is polynomial in nature. In the following subsections, we include an exhaustive analysis for validating the *PM*s in the perspective of bioinformatics research incorporating visual, statistical and biological analysis.

### Visual Validation

**Figure 7:**
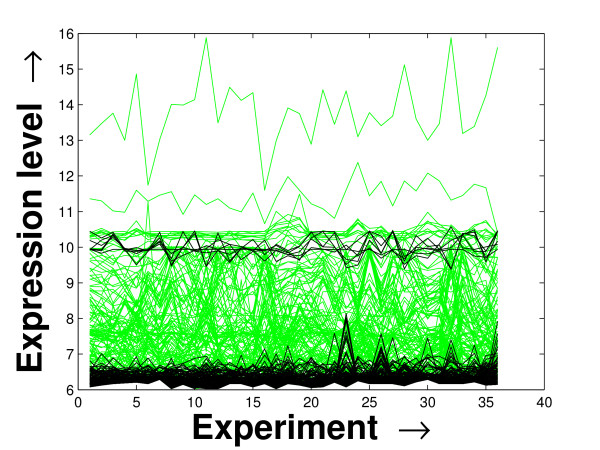
**Expression profile plot of the miRNAs selected and the background set of miRNAs for the schizophrenia dataset**. Expression profile plot of the miRNAs selected in the *PM*s (black) by the proposed method and the background set of miRNAs (green) contained in the schizophrenia dataset.

**Figure 8:**
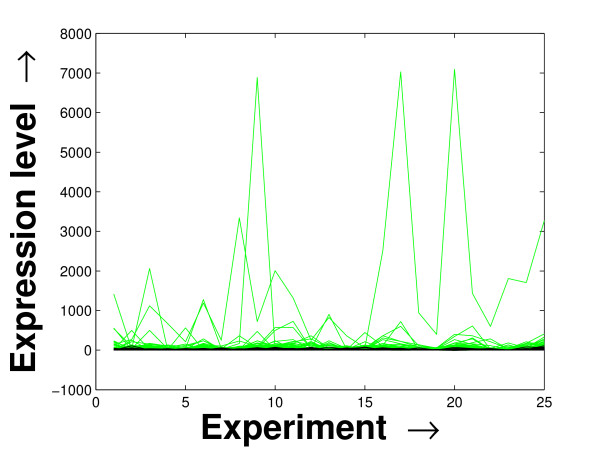
**Expression profile plot of the miRNAs selected and the background set of miRNAs for the tissue-specific dataset**. Expression profile plot of the miRNAs selected in the *PM*s (black) by the proposed method and the background set of miRNAs (green) contained in the tissue-specific dataset.

**Figure 9:**
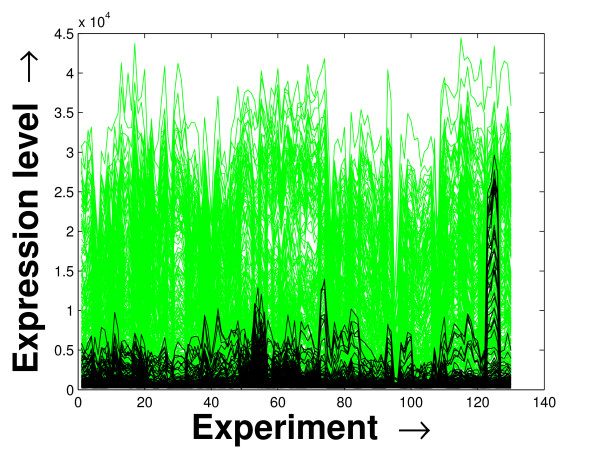
**Expression profile plot of the miRNAs selected and the background set of miRNAs for the stem cell dataset**. Expression profile plot of the miRNAs selected in the *PM*s (black) by the proposed method and the background set of miRNAs (green) contained in the stem cell dataset.

Expression profile plot is a well-known tool for visualizing expression data [6]. A standard expression data contains the expression values over some experiments/conditions (expression vector) for a set of genes/miRNAs. An expression profile plot shows the graphs of the degree of expression values in combination of all the expression vectors over the columns. Thus, a compact expression profile plot (set of the expression values spanning over a compact band) represents a coherent module. The expression profiles of the selected miRNAs in the *PM*s and the background set of miRNAs are plotted in Figures 7, 8 and 9. The proposed method iteratively prepares a few sets of significant miRNAs (*PM*s) from the miRNAs present in the microarray data by mining the constructed FCGs (co-expression networks). Thus, the residual part of the miRNAs, identified as unimportant ones, are kept as the background set. For all the three datasets, the significant fragment of the miRNAs selected by the proposed method spans a compact band of expression levels within the complete band of expression levels of all the miRNAs. More closer two expression levels denote a higher degree of co-expression between the corresponding miRNAs. In case of the schizophrenia dataset, we observe an additional band of selected miRNAs around the expression value 10. This is due to the inherent nature of the proposed mining tool of giving importance to the compactness within the *PM*s over the connectedness between them [20].

**Figure 10:**
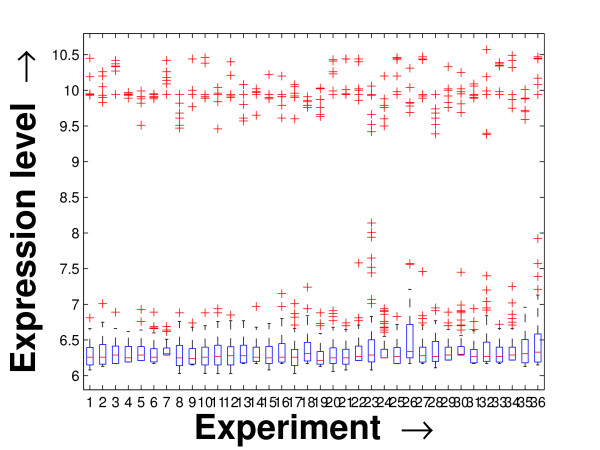
**QDP of the selected set of miRNAs for the schizophrenia dataset. **The Quartile Deviation Plot of the selected set of miRNAs explored by the mining method from the schizophrenia dataset.

**Figure 11:**
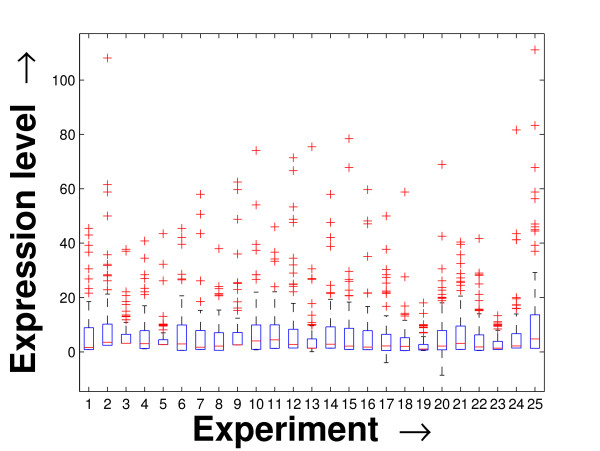
**QDP of the selected set of miRNAs for the tissue-specific dataset**. The Quartile Deviation Plot of the selected set of miRNAs explored by the mining method from the tissue-specific dataset.

**Figure 12:**
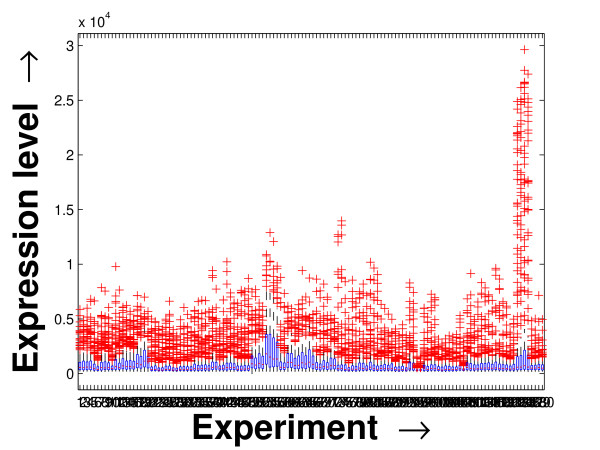
**QDP of the selected set of miRNAs for the stem cell dataset**. The Quartile Deviation Plot of the selected set of miRNAs explored by the mining method from the stem cell dataset.

**Figure 13:**
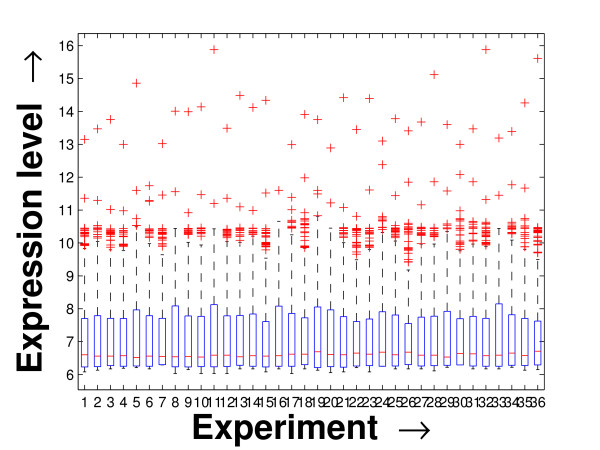
**QDP of the background set of miRNAs for the schizophrenia dataset**. The Quartile Deviation Plot of the background set of miRNAs belonging to the schizophrenia dataset.

**Figure 14:**
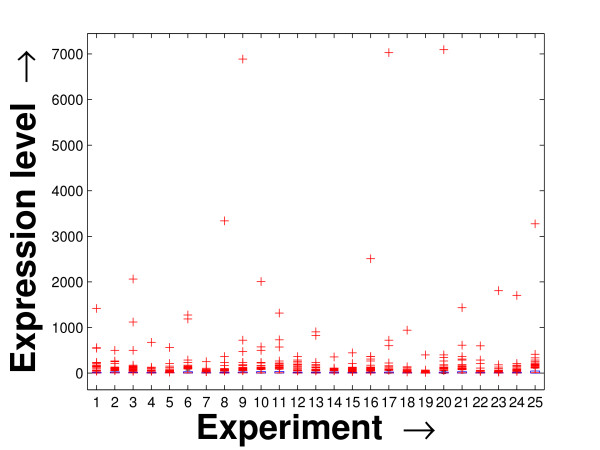
**QDP of the background set of miRNAs for the tissue-specific dataset**. The Quartile Deviation Plot of the background set of miRNAs belonging to the tissue-specific dataset.

**Figure 15:**
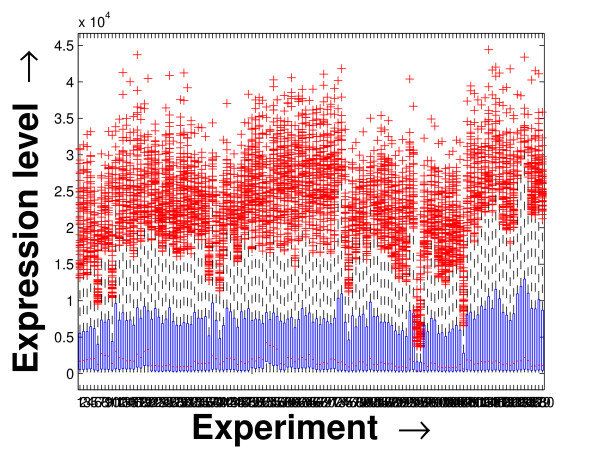
**QDP of the background set of miRNAs for the stem cell dataset. **The Quartile Deviation Plot of the background set of miRNAs belonging to the stem cell dataset.

Performing a rigorous survey, we observed some limitations of the conventional tools (expression profile plot [7], Eisen plot [6]) used for visualizing expression data. These tools are not statistically informative. In particular, the quantitative range of expression values are not observable, deviation in the data can not be expressed and the outliers can not be highlighted through these plots. A novel visual validation plot, referred to as quartile deviation plot (QDP), that can take care of these limitations, has been introduced in this study. The set of expression values of all the miRNAs for a specific experiment (spanning over a single column of the microarray data) is considered as an experiment-specific expression vector. A QDP combines, for each such experiment, the plots of lower quartile, median, and upper quartile values of these expression vectors. The maximum whisker length (in units of interquartile range) is taken as 1.5, which is a default one [23]. The QDPs for all the datasets explored are shown in Figures 10, 11, 12, 13, 14 and 15. Figures 10, 11 and 12 show the QDPs of the miRNAs mined as significant from the three datasets by the proposed method, whereas the Figures 13, 14, and 15 show the QDPs of all the miRNAs present in the datasets. The selected miRNAs, as can be seen from these figures, are relatively more coherent in nature as compared to the complete set. Again from these figures, we can effectively observe the expression pattern (boxplots), expression deviation (height of the boxplots), outliers (plus signs) and also the statistical details (mean values in the boxplots and the whiskers) pertaining the datasets. Thus, the newly proposed QDP tool demonstrates its effectiveness in computational biology.

**Figure 16:**
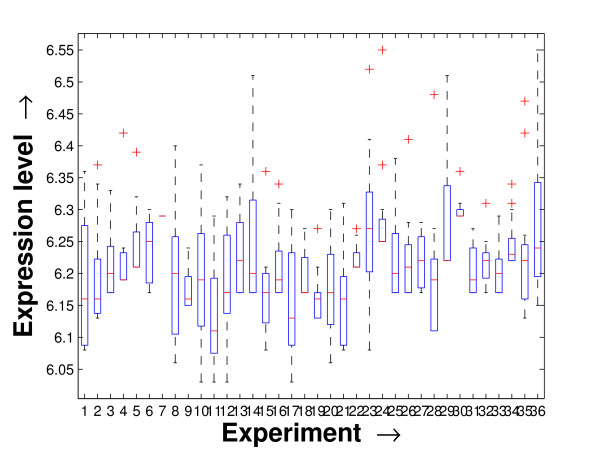
**QDP of the *PM*1 of size 13 found by the proposed method on schizophrenia dataset**. The Quartile Deviation Plot of the *PM*1 of size 13 found by the proposed heuristic mining method applied on the schizophrenia dataset.

**Figure 17:**
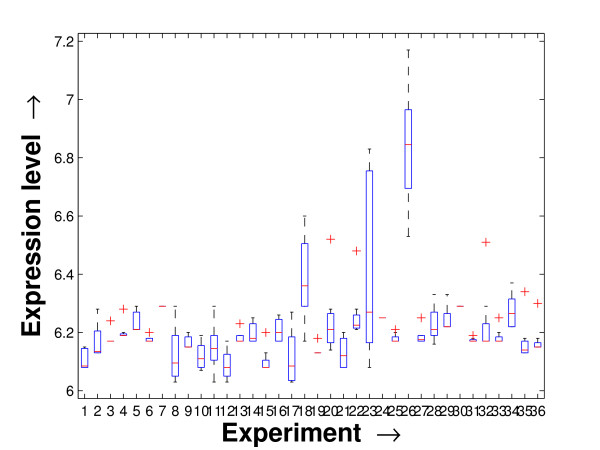
**QDP of the *PM*2 of size 8 found by the proposed method on schizophrenia dataset**. The Quartile Deviation Plot of the *PM*2 of size 8 found by the proposed heuristic mining method applied on the schizophrenia dataset.

**Figure 18:**
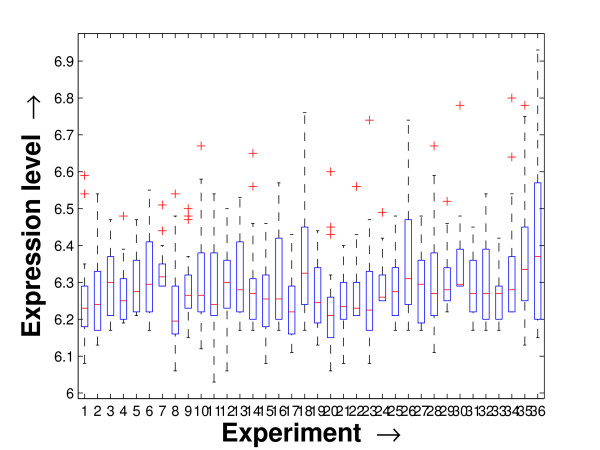
**QDP of the *PM*3 of size 26 found by the proposed method on schizophrenia dataset**. The Quartile Deviation Plot of the *PM*3 of size 26 found by the proposed heuristic mining method applied on the schizophrenia dataset.

**Figure 19:**
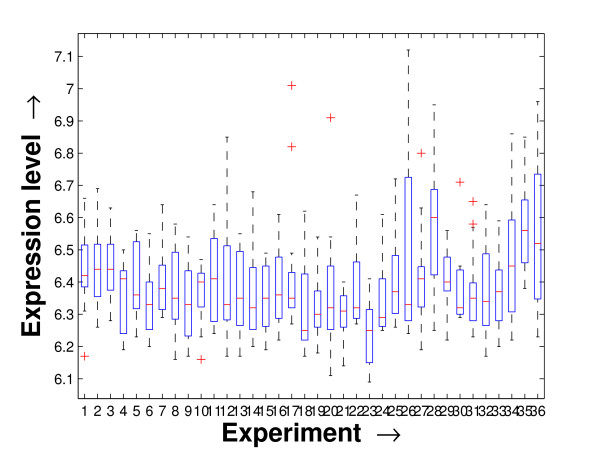
**QDP of the *PM*4 of size 15 found by the proposed method on schizophrenia dataset**. The Quartile Deviation Plot of the *PM*4 of size 15 found by the proposed heuristic mining method applied on the schizophrenia dataset.

**Figure 20:**
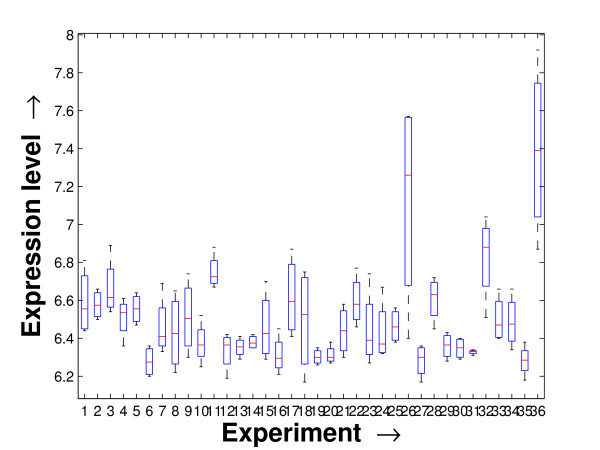
**QDP of the *PM*5 of size 4 found by the proposed method on schizophrenia dataset**. The Quartile Deviation Plot of the *PM*5 of size 4 found by the proposed heuristic mining method applied on the schizophrenia dataset.

**Figure 21:**
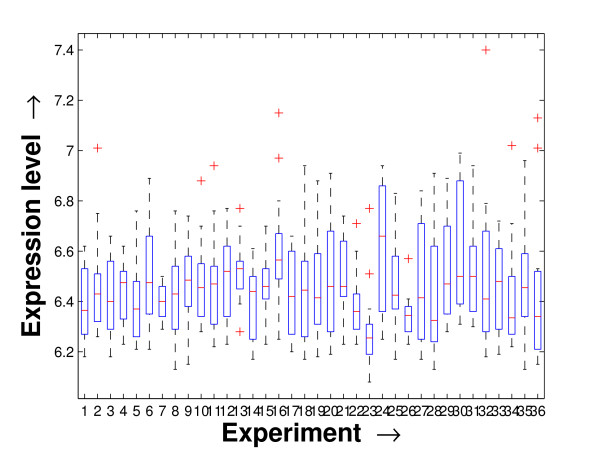
**QDP of the *PM*6 of size 14 found by the proposed method on schizophrenia dataset**. The Quartile Deviation Plot of the *PM*6 of size 14 found by the proposed heuristic mining method applied on the schizophrenia dataset.

**Figure 22:**
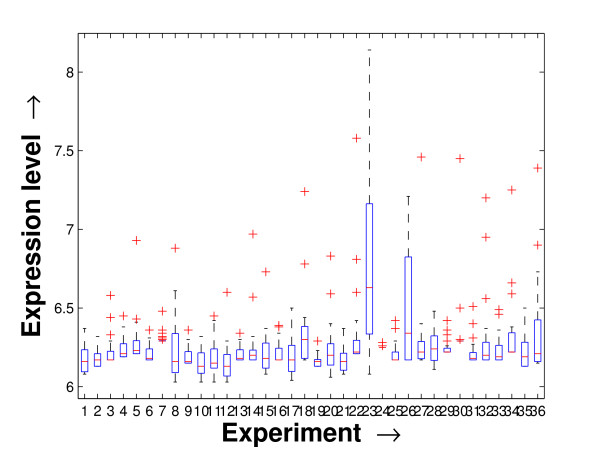
**QDP of the *PM*7 of size 25 found by the proposed method on schizophrenia dataset**. The Quartile Deviation Plot of the *PM*7 of size 25 found by the proposed heuristic mining method applied on the schizophrenia dataset.

**Figure 23:**
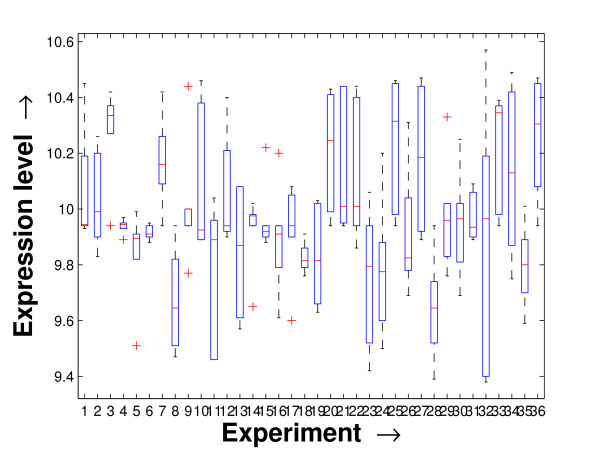
**QDP of the *PM*8 of size 6 found by the proposed method on schizophrenia dataset**. The Quartile Deviation Plot of the *PM*8 of size 6 found by the proposed heuristic mining method applied on the schizophrenia dataset.

To visually validate the degrading coherence within the *PM*s, the expression profile plots (see Additional file 1 section 2.6) and the QDPs of each of the clusters found from the datasets are prepared. Figures 16, 17, 18, 19, 20, 21, 22 and 23 show the plots for the schizophrenia dataset (the plots for the tissue-specific dataset is provided in Additional file 1 section 2.6). As expected, the *PM*s show decreasing order of coherence as they are evolved through the proposed methodology. Moreover, on examining the QDPs more closely, the actual width of the expression band of the *PM*s can be determined for the *PM*s gradually derived by the proposed algorithm.

### Statistical Validation

For the statistical analysis of the *PM*s, we have used a randomized model [3]. Here, a cluster matrix of size *n *× *k *(*n *denotes the number of miRNAs selected in the *PM*s and *k *is the number of *PM*s) is first constructed from the information available about the *PM*s. An element (*i*, *j*) in the cluster matrix is assigned a value "1", if miRNA *i *is found in the *PM j*, otherwise it is set to "0". Depending on the matrix, an *r*-randomized degree preserving model is derived by randomly swapping the edges *r *times for computing the co-occurrence of miRNA pairs by chance. Using the model, the *p*-values (details in Additional file 1 section 2.7) of the co-occurrence of all the miRNA pairs in the *PM*s are computed for all the three datasets. We obtained the values 6.4E-3, 2E-15 and <1E-3 for the schizophrenia, tissue-specific and stem cell datasets, respectively. This shows that the results obtained are not by chance and the *PM*s are statistically significant.

**Figure 24:**
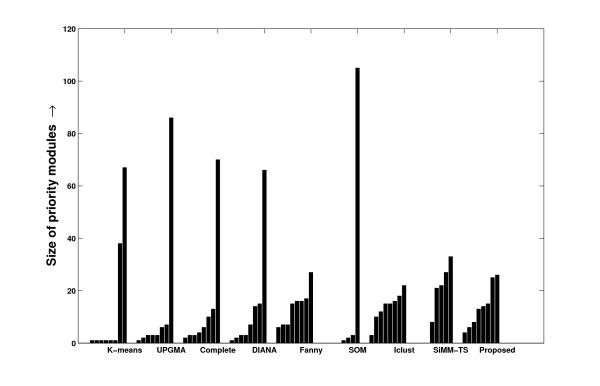
**Distribution of the sizes of the modules identified by various methods from the schizophrenia dataset**. The distribution of the sizes of the modules identified by various clustering algorithms and by the proposed one from the selected set of miRNAs identified from the schizophrenia dataset.

**Figure 25:**
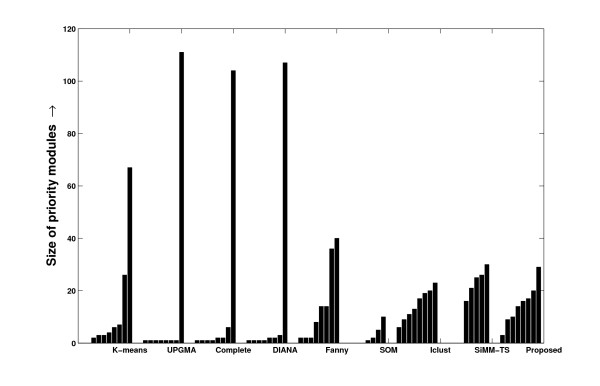
**Distribution of the sizes of the modules identified by various methods from the tissue-specific dataset**. The distribution of the sizes of the modules identified by various clustering algorithms and by the proposed one from the selected set of miRNAs identified from the tissue-specific dataset.

**Figure 26:**
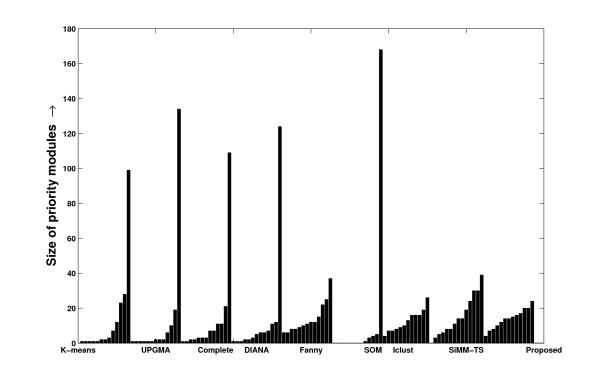
**Distribution of the sizes of the modules identified by various methods from the stem cell dataset**. The distribution of the sizes of the modules identified by various clustering algorithms and by the proposed one from the selected set of miRNAs identified from the stem cell dataset.

The method of finding *PM*s have a close resemblance with the clustering approaches applied to expression data [8,20]. Despite the fact that their motivations differ, a clustering solution ordered in the descending degree of coherence within the clusters can be thought of as a set of *PM*s. So, we include here a comprehensive evaluation of the proposed method with some existing clustering methods in evolving the priority modules. For this purpose, some appreciated clustering methods viz., k-means, average linkage hierarchical (UPGMA) and complete linkage hierarchical clustering from MATLAB, DIANA and Fanny from the R package, Iclust [24] from the author's code, SOM from the standard codes, and SiMM-TS on request from the corresponding author are considered. The algorithm given in Table 1 is written in C language compatible with the gcc compiler in UNIX platform. The comparative results are described in Additional file 1 section 2.8. In Figures 24, 25 and 26, the distribution of the cluster sizes found by various methods are shown. The distribution of the *PM*s derived by the proposed one are found to be comparatively more even in nature.

A current survey [20] classifies the cluster validation measures depending on the clustering criterion into the types – *compactness, connectedness, separation, combinations, stability, preservation of distance*, and *specialized cases*. The current work is motivated from the selection of *PM*s with decreasing compactness. Therefore, a novel internal validation measure to figure out the compactness of the clusters is used here. This compactness measure, the normalized squared error (Σ*NSE*), of a priority mining solution with *k *modules is computed as,

(5)

where |*C*_*i*_| denotes the number of data points in the cluster *C*_*i*_. The Σ*NSE *measure shown in Eqn. (5) normalizes the *SE *values of the clusters with respect to their sizes. This helps to reduce the biased contribution of tiny modules with high compactness in the coherence measure. A lower Σ*NSE *value denotes a higher compactness within the modules. By applying the clustering methods considered in this study on the significant modules found by the proposed method for all the three datasets we found some clusters. After ordering them in the descending order of coherence, we assume them to be *PM*s. After computing the Σ*NSE *values for all the methods to be compared (shown in Table 5), we found that the proposed one is evidently a good one in identifying *PM*s. The sizes of the *PM*s obtained from several approaches like UPGMA or DIANA seems to be irrelevant (modules found with a single miRNA) in the perspective of biology research. They largely fail to identify multiple strong modules and get stuck in finding one large module. Others are better in this sense, specially Fanny and the proposed one, to locate well-distributed coherent modules.

**Table 5:** Comparative Σ*NSE *values

Methods	Schizophrenia dataset	Tissue-specific dataset	Stem cell dataset
K-means	0.9	780.95	5.94E7
Average linkage (UPGMA)	0.81	2069.49	6.62E7
Complete linkage	0.7	1975.34	4.67E7
DIANA	0.71	2107.95	5.02E7
Fanny	0.67	1558.66	4.45E7
SOM	1.22	1126.4	2.46E8
Iclust	12.8	1470.83	1.19E8
SiMM-TS	23.71	1666.38	1.17E8
Proposed	0.64	763.31	4.55E7

Σ*NSE *values computed for different clustering solutions and for the solution derived by applying the proposed method.

### Biological Validation

From a biological perspective, it may be expected that the miRNAs within a single *PM *are regulated by common TFs. To verify this hypothesis, an exhaustive biological investigation has been conducted. Since the complete information related to TF-miRNA regulation is not yet available, we relied on the established knowledge of the conserved TFBSs based on the UCSC hg18 genome assembly [25]. We have used the wgRNA table under the sno/miRNA track of this database (details in Additional file 1 section 2.8) pertaining to the information about the location of miRNAs in the chromosomes. Motivated from an earlier study [3], the region 10 kb upstream of the start of an miRNA sequence is defined as the putative regulatory region of the miRNA assumed to contain the regulatory binding sites. After defining the putative regulatory regions of the miRNAs found in the individual *PM*s, we identified the TFs, which are known to bind to this region, from the tfbsConsSites table under the TFBS Conserved track in the UCSC Table Browser [25]. In this way the list of the miRNA pairs, containing the TFBSs of common regulatory TFs in their putative upstream region, belonging to a single *PM *are accumulated for the study (provided in Additional files 2, 3 and 4 for the schizophrenia, tissue-specific and stem cell datasets, respectively).

All the consecutive *PM*s are exhaustively tested to examine their significance in providing TF-miRNA regulation information found from the three datasets. The number of the miRNAs that are possibly regulated by common TFs by binding to the region upstream of 5' end are given in Table 6 as obtained for all the *PM*s derived from the datasets. For all these datasets, we found very large number of TF-miRNA regulation information for the first few modules. Equivalently for all these datasets, the later modules are found to provide lesser information in this regard. Some of the results of Table 6 may emphasize that some of the initial (generated prior) modules are less important. But, reasonably these are very small modules and therefore cannot capture significant information. Again, such results suggest the appropriate selection of the controlling parameters *δ *and *ξ *for mining biologically more significant results. It may be noted that for *PM *1 found from the schizophrenia dataset, TF information is available for only 20 of the 30 miRNAs. Of these, 16 miRNAs are found having common TFs in their putative 5' UR as per the established results collected from UCSC browser. Similarly, 30 and 42 miRNAs, from a total of 36 and 50 miRNAs, are found to have common such TFs from the total 60 and 57 miRNAs selected in *PM*1 obtained from the tissue-specific and stem cell datasets, respectively. The TF V$AML1 01 is found to bind in the 10 kb 5' UR of a large set of 9 miRNAs (hsa-miR-140, hsa-miR-141, hsa-miR-144, hsa-miR-154, hsa-miR-19a, hsa-miR-432, hsa-miR-488, hsa-miR-496 and hsa-miR-503) which were selected in the most significant module identified from the schizophrenia dataset. Thus their chance of being commonly regulated becomes higher. On examining each such modules, the prioritywise descending ones are found to provide common TFs regulating smaller modules. In fact, none of priority modules generated starting from the fifth ones are found to explore commonly regulated miRNA modules of size higher than three in case of the schizophrenia dataset. Equally promising results are also obtained when the top *PM*s derived from the tissue-specific dataset [11] is analyzed yielding large number of common TFs. For the tissue-specific dataset, some of the TFs were found to possibily regulate upto 4 miRNAs even in the eighth priority module. But the sizes of such commonly regulated miRNA groups are found to be even more larger in the prior modules. We found the two TFs V$AML1 01 and V$FOXO3 01 binding within the 10 kb region of the 5' UR of at least 10 miRNAs for this dataset. Similar observasions are also obtained by examining the list of miRNA pairs having common TFs binding in their 5' UR obtained from the stem cell dataset. The results given in Table 6 for this dataset highlight the prominent significance of the *PM*s up to the third one. the rest of the modules contain minor miRNA groups that may provide week but important regulatory coherence. Thus, the results obtained from all the datasets resembles with the motivation of ordering biologically significant modules and extracting regulation information from them.

**Table 6:** Statistics of the miRNA pairs explored regulated by the common TFs

Priority modules	Schizophrenia dataset	Tissue-specific dataset	Stem cell dataset
*PM *1	589	1074	1585
*PM *2	51	235	148
*PM *3	81	0	211
*PM *4	260	291	2
*PM *5	0	12	8
*PM *6	13	40	30
*PM *7	1	20	110
*PM *8	24	64	13
*PM *9	-	-	0
*PM *10	-	-	0
*PM *11	-	-	13
*PM *12	-	-	0
*PM *13	-	-	73

The number of the miRNA pairs present in the *PM*s found to be regulated by common TFs for all the three datasets.

Since computational analysis of miRNA regulation is still in a nascent stage, such information is biologically significant. The *PM*s provide information, in a compact form, about a set of miRNAs that might be regulated by common TFs. Interestingly, in many cases it has been observed that some miRNAs present in consecutive *PM*s (not in the same one) are associated with same TFs. This might indicate that these miRNAs should have been within a single *PM*, but got separated because of the choice of the density decay constant (*ξ*). Thus an exhaustive sensitivity analysis of the method on *ξ *needs to be carried out in future. Details are shown in Table 6. The assignment of optimal association density threshold (*δ*) value and the density decay constant (*ξ*) play an important role in the selection of significant module by the proposed mining methodology. This parameter, not tuned properly might cause the inclusion of irrelevant miRNAs in the significant module selected or might disrupt the comprehensiveness of this significant module.

### Biological Insight

Biological findings are often biased by probabilistic events. Thus, it becomes important to justify that the findings are not received by chance. To show the biological importance of the information received on TF-miRNA regulation, statistical tests were performed. We have carried out the statistical evaluation of the results obtained for all the datasets. A total of 1019, 1736 and 2193 commonly regulated miRNA pairs are found in the modules received by applying the proposed mining technique on schizophrenia, tissue-specific and stem cell datasets, respectively. Now to verify the significance of this count, modularization solutions have been generated by randomization and the same count has been performed on them. On analyzing them, we received 578, 1027 and 865 commonly regulated miRNA pairs on an average over 10,000 randomized trial runs. The p-values computed are shown in Table 7 for the three datasets. Not a single one of the 10,000 randomized solutions, for all the datasets, are found to exceed the original result in terms of commonly regulated miRNA pair count. These low p-values justify the biological significance of the proposed method in predicting TF-miRNA regulation.

**Table 7:** Computed p-values of the occurrence of commonly regulated miRNA pairs found by the proposed method in the three datasets

Dataset	p-value
Schizophrenia	< 1*E *- 4
Tissue-specific	< 1*E *- 4
Stem cell	< 1*E *- 4

The p-values computed to statistically evaluate the occurrence of commonly regulated miRNA pairs found by the proposed method in the schizophrenia, tissue-specific and stem cell dataset.

A deeper in silico analysis of the *PM*s derived by the heuristic mining procedure sheds light on some important biological results hitherto unexplored. In a recent study [26], the molecular evolution of an miRNA cluster and its paralogs has been reconstructed. This cluster of miRNAs consists of hsa-miR-17, hsa-miR-18, hsa-miR-19a, hsa-miR-19b, hsa-miR-20, hsa-miR-25, hsa-miR-92, hsa-miR-93, hsa-miR-106a, and hsa-miR-106b. To study the co-expression similarity of this set of miRNAs, we investigated the *PM*s that contain these miRNAs from the results of the schizophrenia dataset. Most of these miRNAs are found in separate *PM*s or are pruned out, and therefore, are not co-expressed. Strikingly, although the hsa-miR-19a and hsa-miR-19b are known to be closely related mature sequences (generally represented as hsa-miR-Xa/b/...), yet they are not found in same *PM*s (or even close ones). This might be due to the reason that they are evolutionary clustered. In short, they are not found to be co-expressed although they are paralogs. Therefore, this indicates that the expression profiles might not be dependent on the evolutionary relationship of the miRNAs.

## Conclusion

This paper introduces a novel unsupervised method of exploring commonly regulated modules of human miRNAs by targeting TFs. The method integrates a self-pruning subroutine to discard the portion of the microarray data that might be noisy or insignificant for the particular study. The method has a different motivation from a general clustering approach. It can produce priority-based modules pertaining biological significance. For validating the efficacy of the pruning methodology, a novel tool is devised for visualizing the expression data from a statistical perspective. The results show the generation of a set of *PM*s in the decreasing order of statistical significance. The coherence of these modules is validated with a novel compactness measure. Biologically, with respect to regulation by TFs, this ordering might not be important, even though these *PM*s are found to be effective in the exploration of TF-miRNA regulatory activity. By a deeper analysis, a large number of TFs are identified, which might be regulating multiple miRNAs common to a module. Supporting an earlier study [3], these results might be significant for reconstructing the complex regulatory network that hypothetically exists between TFs and miRNAs. The results also indicate that the miRNAs which are evolutionarily related may not be biologically corregulated.

## Methods

To apply the proposed heuristic mining process, we initially construct an FCG from the microarray data. As this study integrates the concept of FCG, reflecting similarity measure within (0,1], there should be some normalized similarity measure as the fuzzy membership function. Here, a fuzzy membership function, based on the squared Euclidean distance, is used. A commonly used normalization method is performing the zero mean and unit normalization operation (see Additional file 1 section 2.1) on the entire dataset. However, with prior zero mean and unit normalization, the squared Euclidean distance metric coincides with the Pearson correlation coefficient. We employ a novel fuzzy membership function to compute the miRNA-miRNA membership value (relation) in the final FCG.

The proposed fuzzy membership function is based on normalized squared Euclidean similarity computation between two expression vectors *ε*_1 _and *ε*_2_. Suppose, two expression vectors, *ε*_1 _and *ε*_2_, represent the expression values of the two vertices *v*_1 _and *v*_2 _(or equivalently the miRNAs corresponding to *v*_1 _and *v*_2_), then the fuzzy membership value of the edge (*v*_1_, *v*_2_) is defined as,

(6)

In Eqn. (6), *ε*_1*i *_represents the *i*^*th *^element of the expression vector *ε*_1 _and *NF *denotes a normalization factor that is calculated as .

The FCG to be explored is prepared using the aforesaid measure. Once the FCG is prepared they can be equivalently considered as a co-expression network. The proposed mining method produces a set of N-vertexlets (groups of miRNAs which we call *PM*s) by stepwise pruning of the constructed FCG until a stopping criterion is reached.

The proposed mining methodology is given in formal steps in Table 1. This complete process is followed by a post-processing technique. The basic algorithm efficiently groups the miRNAs in the descending order of coherence and prunes out the insignificant residual part. It takes an miRNA co-expression network (in the form of FCG) and the two controlling parameters a lower density threshold and a density decay constant as inputs. Staring from the zeroth time point (*t *= 0), at each iteration (time point) the algorithm discovers the largest *PM *(largest dense N-vertexlet) in the current co-expression network. This (step 4) is done by using an algorithm proposed in a recent work to identify largest N-vertexlets from a scale-free graph [17].

The process of identifying the largest *PM *works like this:

1. For every single vertex in the FCG a neighboring list of vertices is prepared. This contains the series of vertices in their descending order of fuzzy membership value with respect to the corresponding vertex.

2. The vertex having the maximum association density with respect to the remaining ones is selected as the seed vertex.

3. The seed vertex is expanded heuristically by weighted combination of the neighboring list until a threshold of association density (here *δ*_*t*_) is reached.

4. The final expanded list provides the largest *PM*.

The selected largest *PM *obtained using the above subroutine is extracted from the original network and the association density is decayed. The decay of density does not occur linearly, rather, it is done inspired by an approach similar to simulated annealing associating a decay constant *ξ*. This decayed density and the residual network are taken as the current density and current network, respectively, in the subsequent iteration. The self-pruning is continued until the lower density threshold is reached and the left-out network is treated as the insignificant subpart of the original network. On completion of the iterations, the number of *PM*s is returned by the variable *t*. The output is produced in the form of a finite set of *PM*s. From the entire set of *V *miRNAs,  miRNAs are mined as significant part and the left portion is pruned out. Thus, it statistically integrates a noise-pruning characteristic to produce accurate results.

Subsequent to this mining procedure a post-processing routine is performed on the final set of *PM*s {} produced as the output. These *PM*s are selected as a set of initialized modules and the centers of these modules are computed. With respect to all the miRNAs, the modules are reconstructed by associating each miRNA to a closer module center. Again, the module centers are computed for the reconstructed modules and the same process is iterated. This finally produces the modules of miRNAs of importance.

Supplementary materials along with the datasets are available at the webpage of the corresponding author: .

## Authors' contributions

SB and MB jointly carried out the literature survey, pre-work planning and algorithmic development. Both of them conceived of the study and MB implemented the algorithms. Both SB and MB contributed to prepare the draft of the manuscript. SB corrected the draft version and incorporated significant revisions. Both the authors read and approved the final manuscript.

## Supplementary Material

Additional file 1 **Supplementary details**. The supplementary details elaborates the employed methods, the results received by applying them and the dataset description. The entire findings on the TF-miRNA regulation over the datasets used are accumulated in tabular form and given in the supplementary material. The to-the-point methodological descriptions are also given with full theoretical details in this file. Click here for file

Additional file 2 **TF-miRNA regulation information obtained for the schizophrenia dataset**. It accumulates the list of the miRNA modules, which have TFBSs of some common TFs in their 10 kb 5' UR, found in all the *PM*s identified by the proposed method from the schizophrenia dataset. Click here for file

Additional file 3 **TF-miRNA regulation information obtained for the tissue-specific dataset**. It accumulates the list of the miRNA modules, which have TFBSs of some common TFs in their 10 kb 5' UR, found in all the *PM*s identified by the proposed method from the tissue-specific dataset. Click here for file

Additional file 4 **TF-miRNA regulation information obtained for the stem cell dataset**. It accumulates the list of the miRNA modules, which have TFBSs of some common TFs in their 10 kb 5' UR, found in all the *PM*s identified by the proposed method from the stem cell dataset. Click here for file
