# Supplementary material for: Analyzing miRNA co-expression networks to explore TF-miRNA regulation
Source: BMC Bioinformatics. 2009 May 28;10:163. doi: 10.1186/1471-2105-10-163 (PMC2707367; doi:10.1186/1471-2105-10-163)
Supplement: Additional file 1 — Supplementary details. The supplementary details elaborates the employed methods, the results received by applying them and the dataset description. The entire findings on the TF-miRNA regulation over the datasets used are accumulated in tabular form and given in the supplementary material. The to-the-point methodological descriptions are also given with full theoretical details in this file. [file 1471-2105-10-163-S1.pdf]

Analyzing miRNA co-expression networks to explore TF-miRNA regulation (Supplementary details)

Sanghamitra Bandyopadhyay and Malay Bhattacharyya
Machine Intelligence Unit, Indian Statistical Institute,
203 B. T. Road, Kolkata - 700108, India
E-mail:{sanghami, malay_r}@isical.ac.in

March 19, 2009

Introduction

Microarray analysis of microRNAs (miRNAs) is a high throughput method for studying miRNA expression in cultured cells or tissues. The three datasets used in this analysis are the resultant of three different microarray experiments using human (*Homo Sapience*) miRNAs. We describe the materials and methods employed in the study in the sections ahead.

1 Materials

Three microarray datasets are experimented in this study. One of them comprises the expression values of schizophrenia patient-specific miRNAs [Perkins *et al.*, 2007] while the others contain expression values of tissue-specific miRNAs [Baskerville, 2005] and stem cell-based expression profiling for different subjects [Laurent *et al.*, 2008]. These three are described hereunder.

1.1 Schizophrenia Dataset [Perkins *et al.*, 2007]

This dataset contains the expression values of postmortem human brain tissues, obtained from the Harvard Brain Tissue Resource Center, consisting of frozen blocks (300-500 mg/block) from the postmortem prefrontal cortex (Brodmann area nine from 15 individuals with schizophrenia and 21 unaffected comparison subjects). The subjects taken were free of neurodegenerative pathology and the tissues were groupmatched for gender, age, postmortem interval, and hemisphere.

The dataset was prepared through a microarray experiment where oligonucleotide probes were synthesized in duplicate for 264 miRNAs antisense to the mature sequence. The mature sequences were verified from the reported results accumulated in the Sanger miRNA registry. All arrays were prepared from the same batch using the conventional hybridization process. The microarray analysis started with the extraction of data from the GPR files and the data points having foreground values lower than 1.5 times of the local background were eliminated. Following this elimination, the probes from which greater than 40% of the data points were removed, were discarded out of the analysis. This pre-processing reduced the dataset comprising a total of 239 miRNAs. After the background subtraction all the data were log-transformed and the missing values were estimated using the well-known k-NN [Troyanskaya *et al.*, 2001]. The data were normalized using rank

invariant normalization for comparison across the samples. The per-sample mean of the two rank invariant normalized probes was used for the analysis. Univariate calculations of differential expression were estimated using two-class (unpaired test) Statistical Analysis of Microarrays through 500 permutations with an FDR of 5%.

Thus, the final dataset includes the expression values received over 36 experiments for 239 miRNAs. The statistical information pertaining the microarray data is - minimum expression value = 6.03, maximum expression value = 15.88, average expression value = 7.27, standard deviation of the expression values over the entire dataset = 1.38.

1.2 Tissue-specific Dataset [Baskerville, 2005]

This dataset includes the global expression of miRNAs found in various human tissues. The probes used in the microarray experiment were based on DNA oligonucleotides. Based on the techniques developed earlier to clone and sequence the populations of small RNAs, hybridization and the processing of the arrays were performed. A pilot array was used to explore the hybridization conditions and the array design options during the study. The experimentation achieved expression profiling of 175 miRNAs taken from 24 different human organs and the HeLa S3 cell line. Ongoing research physically identifies some of these miRNAs in brain, lung, liver, spleen, muscle, and bone marrow having highly specific expression in these organs.

There are 43 missing values out of the total 4375 values ($\sim$ 0.98%) in the complete dataset. These missing values are imputed by the BPCA method [Brock *et al.*, 2008]. The statistical information pertaining the microarray data is - minimum expression value = 0.45, maximum expression value = 7095.17, average expression value = 32.33, standard deviation of the expression value over the entire dataset considered = 221.28. These are after the estimation of the missing values.

1.3 Stem Cell Dataset [Laurent *et al.*, 2008]

This microarray-based miRNA expression profiling was performed using a novel modification of the high-throughput gene expression profiling methodology of assays. This combines cDNA-mediated annealing, selection, extension and ligation assay. It applied a solid-phase primer extension (after target hybridization) to enhance the discrimination among homologous miRNA sequences. In addition, PCR with universal primers was used to amplify all targets prior to array hybridization. One specific assay oligonucleotide was designed for each miRNA, consisting of three parts: at the 5' end was a universal PCR priming site; in the middle was an address sequence, complementary to a corresponding capture sequence on the array; and at the 3' end was a miRNA-specific sequence. Pooled assay oligonucleotides corresponding to the human miRNAs are first annealed to cDNA. An allele-specific primer extension step is then carried out; the assay oligonucleotides are extended only if their 3' bases are complementary to their cognate sequence in the cDNA template. The extended products are then amplified by PCR using common primers, of which one is fluorescently labeled, and hybridized to a microarray bearing the complementary address sequences.

A subset of this dataset is used in this study. The extracted dataset includes 130 columns over the 439 human miRNAs without any missing values. In this expression data, minimum expression value = 0, maximum expression value = 29632.83, average expression value = 982.18 and standard deviation of the expression values over the entire dataset = 1067.54.

2 Methods

The methods employed in the study are described in detail in the following subsections.

2.1 Proximity Measure

The zero mean and unit normalization of an expression vector (a row vector) $E = [E_1 E_2 \ldots E_n]$ of dimension n is computed as,

$$\mathcal{E} = \frac{E - [\bar{E}]}{||E||}. \tag{1}$$

Note that, the normalization done using Eqn. (1) turns the expression vector E into a unit vector $\mathcal{E}$ ($||\mathcal{E}|| = 1$). Further applying squared Euclidean measure on this normalized data maps the co-expression values within $[-1, 1]$. But, with this zero mean unit normalization the squared Euclidean distance metric coincides with the Pearson correlation coefficient. As the study intends to prepare a co-expression network, a correlation measure will not therefore fit here as appropriate. Moreover, the study is based on a Fuzzy Complete Graph (FCG) that reflects the degree of fuzzy membership values (similarity) between the vertices. Thus, the necessity of a novel proximity measure becomes apparent here.

2.2 Sensitivity Analysis

The assignment of optimal association density threshold (δ) value and the density decay constant (ξ) play an important role in the selection of significant module by the proposed mining methodology. This parameter, not tuned properly might cause the inclusion of irrelevant miRNAs in the significant module selected or might disrupt the comprehensiveness of this significant module. Naturally, the sensitivity of parameters in an unsupervised method is guided by the data itself. With this prior knowledge, we have observed the distribution of fuzzy membership values within an FCG to get an indication of threshold of the association density. Two kinds of histogram plots have been prepared for both the FCGs derived from the datasets. The first one is shown in the main paper whereas the other is shown in Fig. S1.

In the first kind of histogram (see main paper), the average fuzzy membership values of all the miRNAs are computed with respect to the other miRNAs and its histogram is prepared. These are column-specific values. Whereas, in the other one (Fig. S1), for each experiment included in the dataset, the histogram of the fuzzy membership values of all the miRNAs with respect to the others are combined in a single plot. This highlights the distribution of fuzzy membership values of all the miRNA pairs over the entire dataset. From both of these, we can follow that for higher δ values we get higher associativity within an miRNA module. On observing Fig. S1, one can easily note the long tail of miRNA connectivity for lower δ values. By the careful judgement of these plots, we selected the Association Density threshold to be 0.95, 0.99 and 0.93 for the patient-specific, tissue-specific and stem cell datasets respectively. To care about the noise that might be present in the data, the parameters have been tuned to slightly higher values from that of realizable by perception from the plots.

2.3 Silhouette Index Measure for Priority Modules

For quantitatively testing the significance of the priority modules (PMs) found by the proposed method (for various δ values) and some of the well-known clustering approaches, the Silhouette index [Rousseeuw, 1987], a well-known cluster validity index, has been computed. The Silhouette index can measure the degree of compactness within a single PM and its separation from the set of other PMs (assuming it to be the second cluster) [Bandyopadhyay *et al.*, 2008]. The distance metric used is the squared Euclidean distance. The intercluster separation is calculated as,

$$X = \sum_{v_j \in V-C} \frac{||v_i - v_j||}{|V - C|}. \tag{2}$$

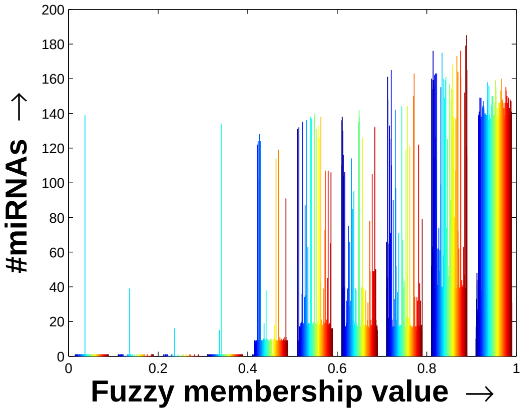

(I)

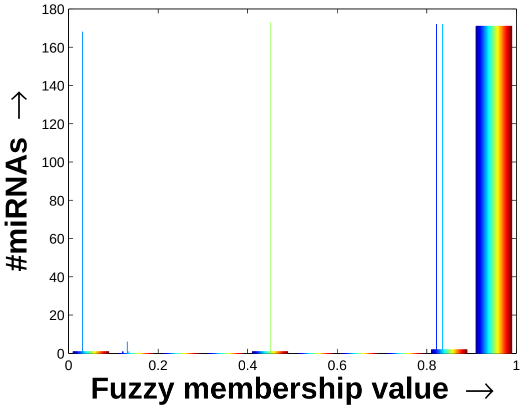

(II)

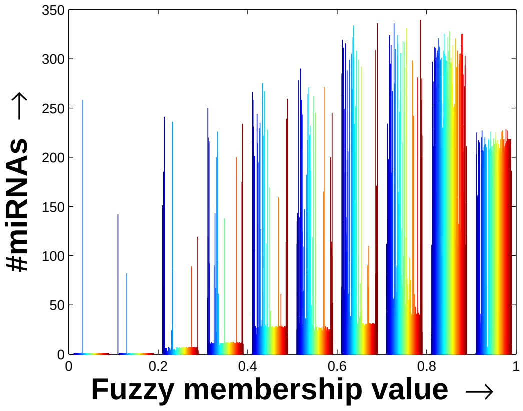

(III)

Figure S1. Histogram of the total similarity values in FCG constructed from (I) schizophrenia dataset, (II) tissue-specific dataset and (III) stem cell dataset.

Again, the intracluster variance is calculated as,

$$Y = \sum_{v_j \in C - \{v_i\}} \frac{||v_i - v_j||}{|C - 1|}. \tag{3}$$

Using Eqn. (2) and Eqn. (3), the average Silhouette width of the cluster C, $SI_{C/V}$, with respect to the complete reference set V, is calculated as,

$$SI_{C/V} = \frac{1}{|C|} \sum_{v_i \in C} \frac{X - Y}{\max\{X, Y\}}. \tag{4}$$

The value of $SI_{C/V}$ ranges within [-1,+1], with higher values indicating better clustering result.

2.4 Validation of Pruning Phase

A clique is defined to be a complete subgraph of a graph. The vertices within a clique is therefore fully associative with each other. So, by exploring the largest clique sizes we have tried to verify the comprehensiveness of the size of the significant modules selected from the patient-specific and tissue specific datasets by the proposed method. For this purpose, the two FCGs, derived from the two datasets, have been mapped initially into two unweighted graphs. This is done by removing those edges having fuzzy membership values less than 0.95, 0.99 and 0.93 from the complete graphs for the three datasets respectively. Following an upper bound of a clique given in [Amin *et al.*, 1972], we have computed the upper bound of clique sizes of the unweighted graphs derived from the FCGs. According to this work [Amin *et al.*, 1972], the clique size $\gamma(G)$ of a graph G is upper bounded by the following relation,

$$\gamma(G) \leq N_{\leq -1} + 1. \tag{5}$$

In Eqn. (5), $N_{\leq -1}$ denotes the number of eigenvalues, of the adjacency matrix of G, which are no larger than -1.

2.5 Results on Gene Expression Data

For verifying the scalability of the proposed mining method, we have applied it on a larger gene expression dataset. The dataset contains 6167 yeast genes and their expression profiles over a total of 52 time points of DNA damage. The missing values of the dataset were imputed with BPCA [Brock *et al.*, 2008] and the final FCG is prepared by computing the co-expression values. The parameters of the mining algorithm were set to $\delta = 0.6$ and $\xi = 0.01$. The distribution of the sizes of the PMs found by the proposed heuristic mining method is shown in Fig. S2(II). As for the miRNA co-expression networks, here too we found that the statistical coherence within these modules are very high. The expression profile plot of the selected significant 216 genes and the background set of genes are shown in Fig. S2(I). We found a total of 25 PMs, with a few of these being small in size. Specially, the first few are very small modules but they have very high coherence which decreases in the later ones.

2.6 Visual Validation

Expression profile plot is a well-known tool for the visualization of expression data [Eisen *et al.*, 1998]. It shows the plots of the degree of expression values of all the expression vectors in combination. Thus, a compact expression profile plot (set of the expression plots spanning over a compact band) represents a

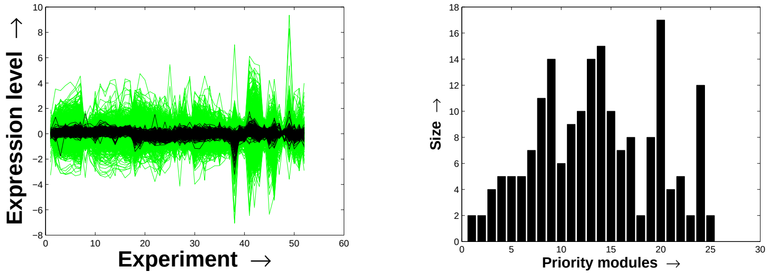

(I) (II)

Figure S2. (I) Expression profile plot of the PMs found by the proposed heuristic mining method applied on the gene expression data where the significant genes selected are shown in black and the left out portion is shown in green, (II) Distribution of the sizes of the PMs found by the proposed heuristic mining method applied on the gene expression data.

Table S1 A cluster matrix

miRNA	$PM1$	$PM2$	$PM3$	...	PMk
hsa-miR-1	0	1	0	...	0
hsa-miR-2	0	0	0	...	0
hsa-miR-3	1	0	0	...	0
...	...	...	...	...	...
hsa-miR-n	0	0	0	...	1

coherent module. To highlight the decreasing coherence the PMs, the expression profile plots of these clusters are shown in Fig. S3 and Fig. S4.

Here, a novel visual validation tool, namely the quartile deviation plot (QDP), has been used to illustrate the effectiveness of the pruning method. This tool incorporates many statistical information in the plot, not only representing the level of expression values. A QDP is a combination of some boxplots. It prepares the boxplot of the expression values over each experiment. Such a columnwise boxplot of the entire dataset reflects the lower quartile, median, and upper quartile values of an experimental column. These boxplots of all the experiments (be it patient-specific or tissue-specific) are then combined to prepare the final plot. The maximum whisker length, in the units of interquartile range, is taken to be 1.5, which is a default one [McGill *et al.*, 1978]. The QDPs of the priority clusters are shown in the main paper.

2.7 Co-occurrence p-value Computation

To validate a mining solution in the form of a set of PMs, it is worth exploring the probability of receiving the result by chance. So, the computation of p-value is of importance here. Earlier, for similar cases a randomization model that preserves the degree distribution in a network by edge-swapping has been effectively used [Shalgi *et al.*, 2007]. We construct here a cluster matrix, of size $n \times k$, where n denotes the number of miRNAs pruned from the complete set and k is the number of PMs. The element in the position (i, j) is "1" if miRNA i is found in PM j, otherwise "0". One such example of a cluster matrix is shown in Table S1.

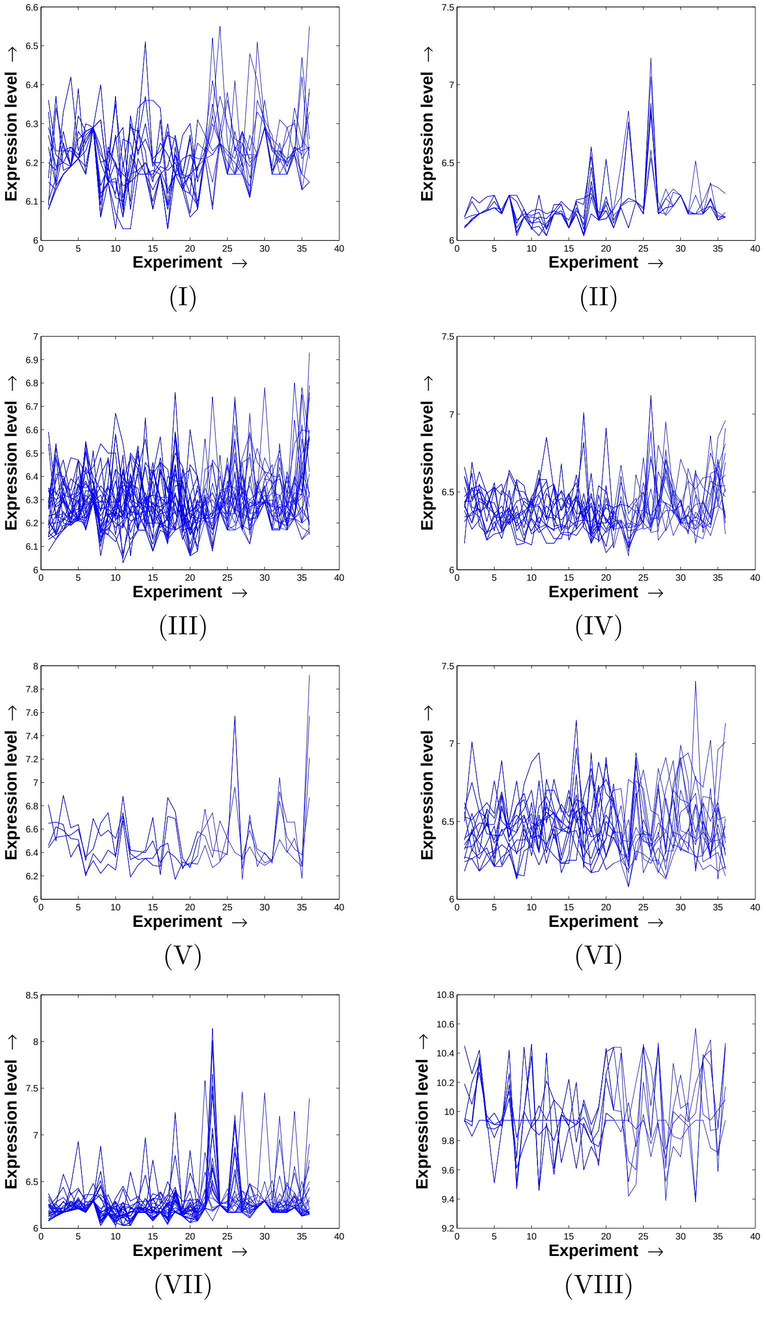

Figure S3. Expression profile plot of the PMs found by the proposed heuristic mining method on schizophrenia dataset (I) $PM1$ of size 13, (II) $PM2$ of size 8, (III) $PM3$ of size 26, (IV) $PM4$ of size 15, (V) $PM5$ of size 4, (VI) $PM6$ of size 14, (VII) $PM7$ of size 25, (VIII) $PM8$ of size 6.

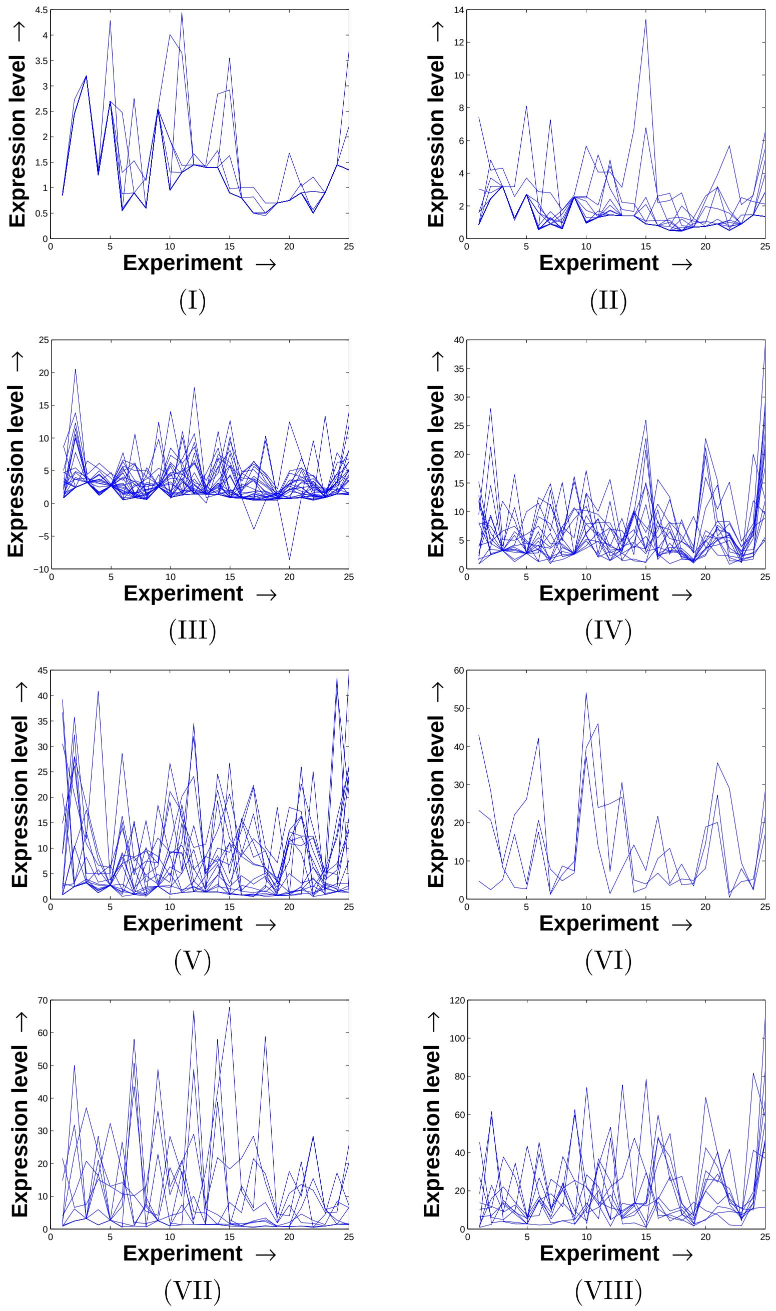

Figure S4. Expression profile plot of the PMs found by the proposed heuristic mining method on tissue-specific dataset (I) $PM1$ of size 13, (II) $PM2$ of size 8, (III) $PM3$ of size 26, (IV) $PM4$ of size 15, (V) $PM5$ of size 4, (VI) $PM6$ of size 14, (VII) $PM7$ of size 25, (VIII) $PM8$ of size 6.

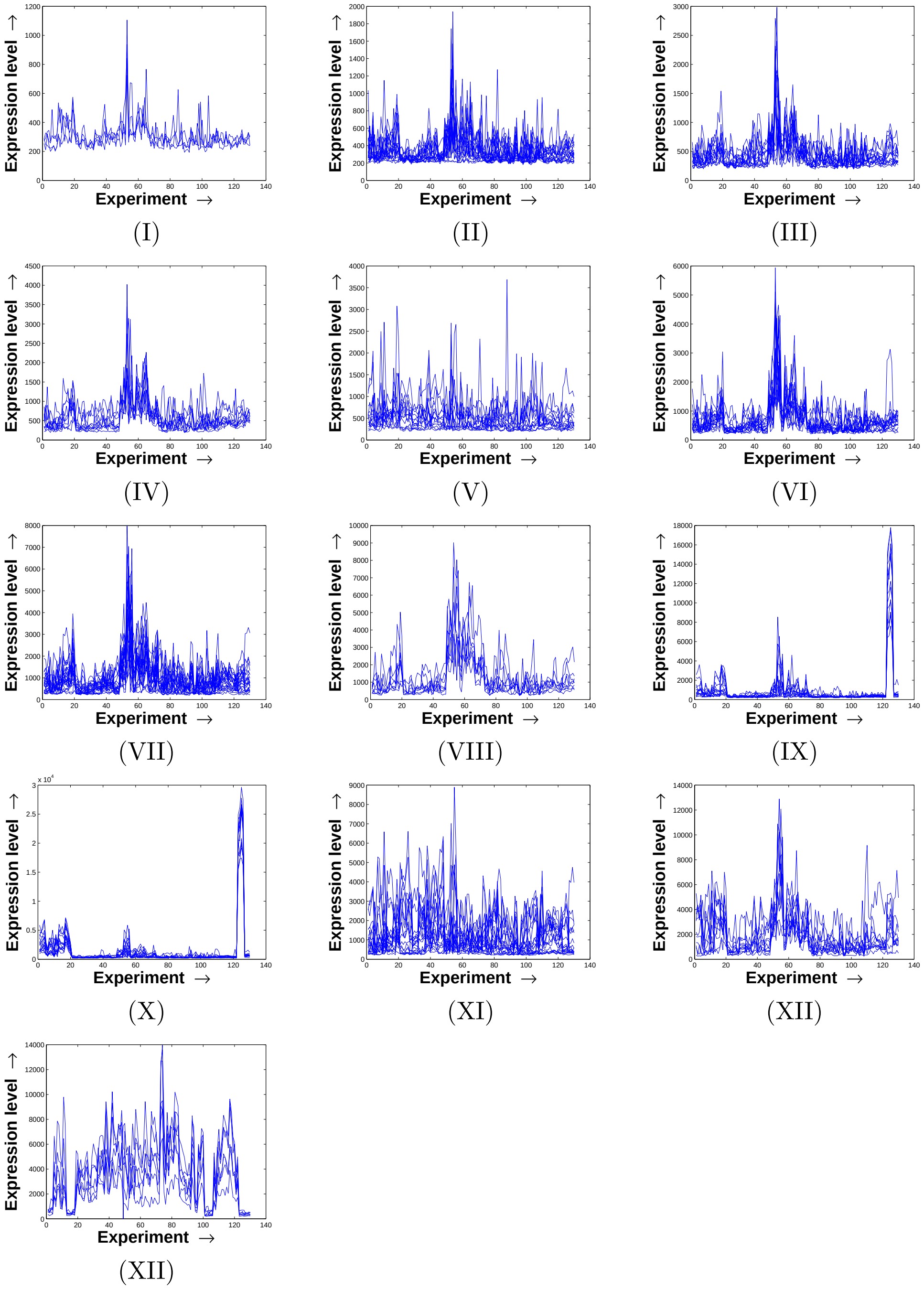

Figure S5. Expression profile plot of the PMs found by the proposed heuristic mining method on stem cell dataset (I) $PM1$ of size 4, (II) $PM2$ of size 20, (III) $PM3$ of size 16, (IV) $PM4$ of size 10, (V) $PM5$ of size 14, (VI) $PM6$ of size 17, (VII) $PM7$ of size 24, (VIII) $PM8$ of size 8, (IX) $PM9$ of size 14, (X) $PM10$ of size 15, (XI) $PM11$ of size 20, (XII) $PM12$ of size 12, (XIII) $PM13$ of size 7.

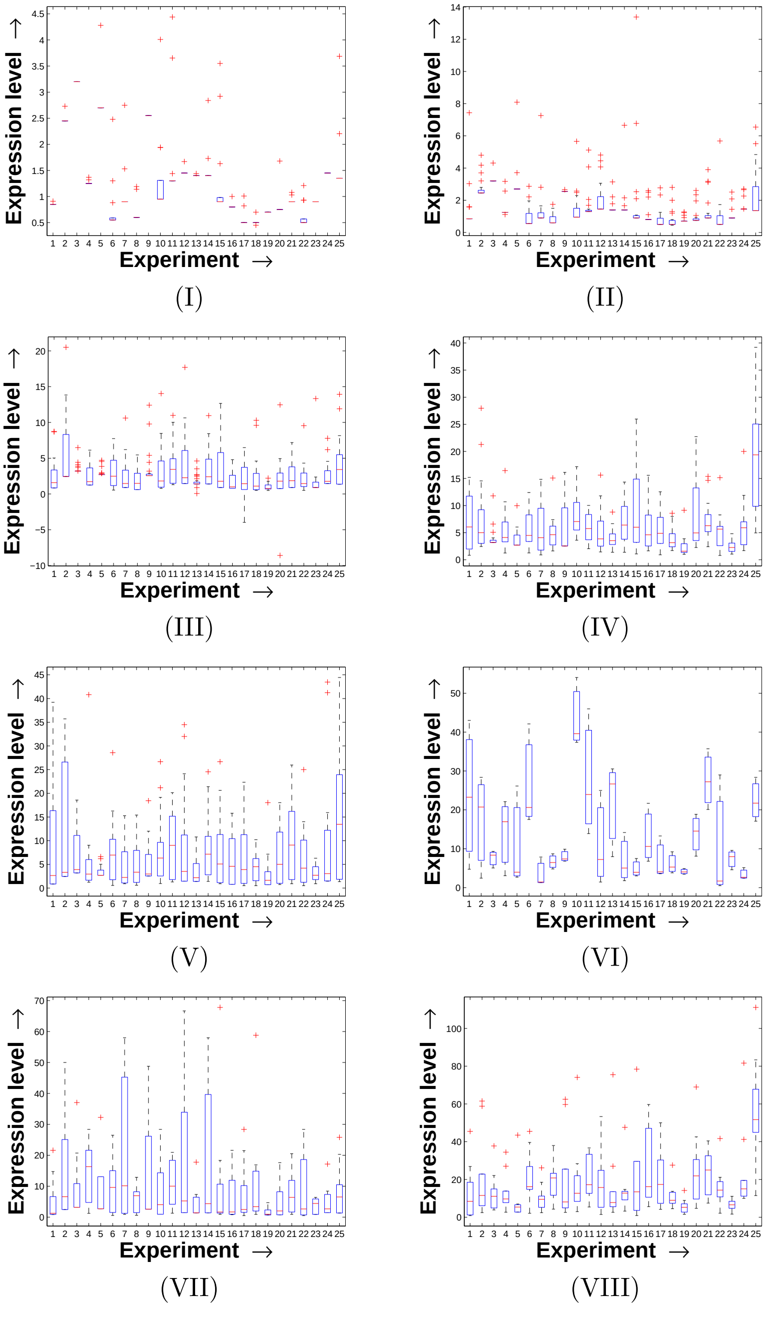

Figure S6. QDP of the PMs found by the proposed heuristic mining method on tissue-specific dataset (I) $PM1$ of size 13, (II) $PM2$ of size 8, (III) $PM3$ of size 26, (IV) $PM4$ of size 15, (V) $PM5$ of size 4, (VI) $PM6$ of size 14, (VII) $PM7$ of size 25, (VIII) $PM8$ of size 6.

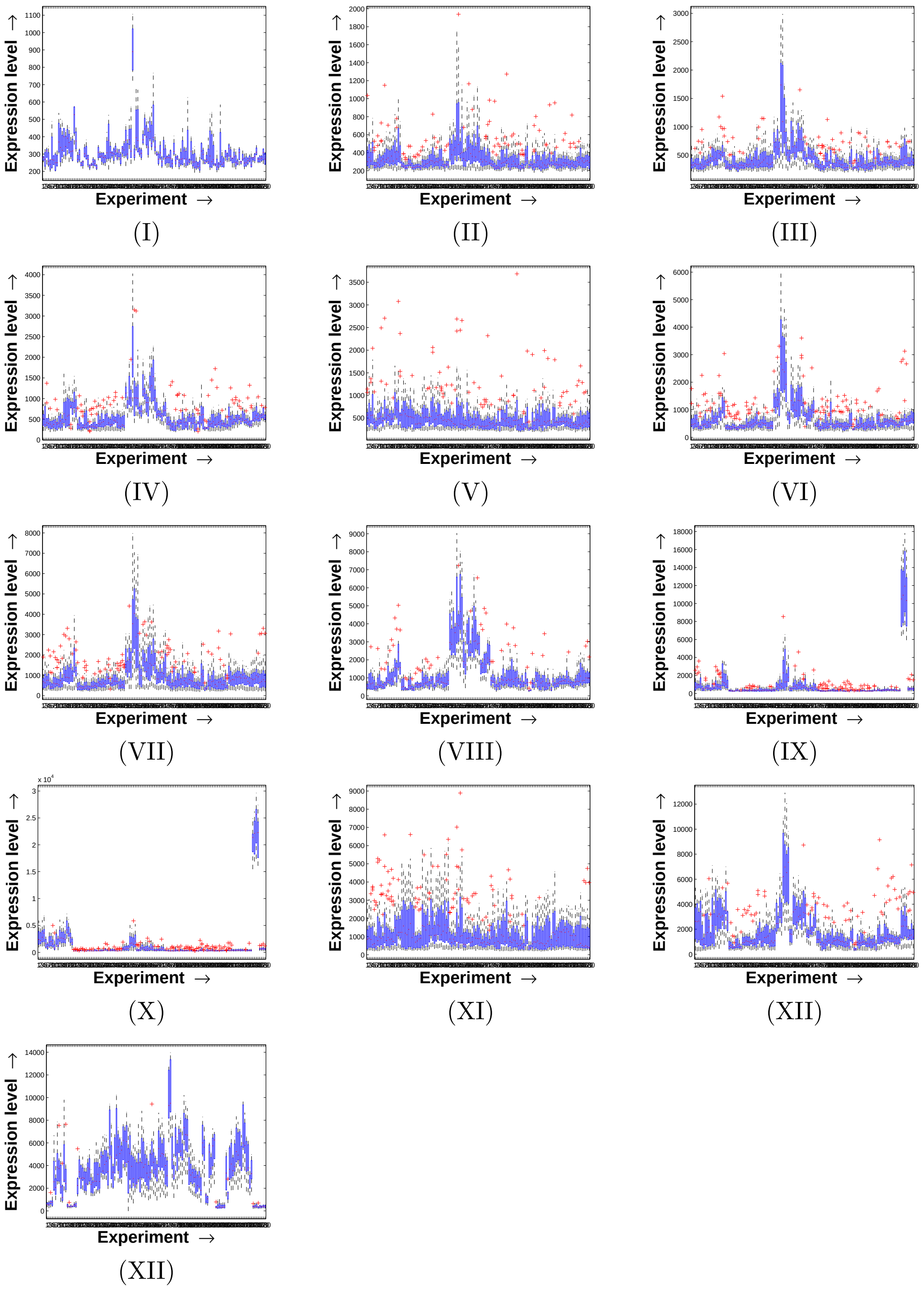

Figure S7. QDP of the PMs found by the proposed heuristic mining method on stem cell dataset (I) $PM1$ of size 4, (II) $PM2$ of size 20, (III) $PM3$ of size 16, (IV) $PM4$ of size 10, (V) $PM5$ of size 14, (VI) $PM6$ of size 17, (VII) $PM7$ of size 24, (VIII) $PM8$ of size 8, (IX) $PM9$ of size 14, (X) $PM10$ of size 15, (XI) $PM11$ of size 20, (XII) $PM12$ of size 12, (XIII) $PM13$ of size 7.

Now, the values of the matrix are altered repeatedly to make it random. In this randomization subroutine, the values are actually swapped randomly between two miRNA pairs to keep the distribution of the values same in the matrix. After preparing the final random matrix, the co-occurrence of the miRNA pairs are computed in the matrix. Based on this randomized result and the co-occurrence count of the original matrix, the p-value is computed. Suppose, the co-occurrence of miRNAs are found n times out of a total N cases of the cluster matrix and computing in the randomized matrix, the count is found to be e times out of a total N cases. Then, the hypergeometric p-value is computed as,

$$p\text{-value} = \sum_{i=n}^{N} \frac{\binom{e}{i}\binom{E-e}{N-i}}{\binom{E}{N}}. \tag{6}$$

A poor p-value computed from a solution establishes the truth of not receiving the result by chance.

2.8 Transcription Analysis

For the transcription regulatory analysis within the *PM*s found by the proposed method, the well-known UCSC Table browser [Karolchik *et al.*, 2004] is used. This manually updated database contains many established results in tabular format. The browser helps to retrieve the data associated with a track in text format and to calculate the intersections between various tracks. We have mainly used two tables. The first one accumulates the locations of the human miRNAs in the chromosomes including the information on start sites and end sites. The table is downloadable from the UCSC Table browser [Karolchik *et al.*, 2004] with the following options:
clade: Mammal,
genome: Human,
assembly: March. 2006 (up to date assembly),
group: Genes and Gene Prediction Tracks,
track: sno/miRNA,
table: wgRNA,
identifiers (names/accessions): user defined.

After accumulating the data on the locations of miRNAs for both the datasets, we have searched for the transcription factors (TFs) binding in the 10kb upstream region of the individual miRNAs. The complete table has been downloaded from the UCSC Table browser [Karolchik *et al.*, 2004] by setting the options as follows:
clade: Mammal,
genome: Human,
assembly: March. 2006 (up to date assembly),
group: Regulation,
track: TFBS Conserved,
table: tfbsConsSites,
region: user defined (genome).

This track shows microRNA target sites in the defined regions. Now, by studying the retrieved tables, we accumulate the results finally into some tables. These tables show the list of TFs which are found in the $5'$ untranscribed region (UR) of more than one miRNAs common to a single *PM*. Thus, by unsupervised means we can find out common transcription targets of miRNAs which helps to reconstruct the regulatory network between miRNAs.

References

[Amin *et al.*, 1972] Amin,A.T. and Hakimi,S.L. (1972) Upper bounds on the order of a clique of a graph, *SIAM J. Appl. Math.*, **22**, 569-573.

[Bandyopadhyay *et al.*, 2007] Bandyopadhyay,S. *et al.* (2007) An improved algorithm for clustering gene expression data, *Bioinformatics*, **23**, 2859-2865.

[Bandyopadhyay *et al.*, 2008] Bandyopadhyay,S. and Bhattacharyya,M. (2008) Mining the Largest Dense N-vertexlet in a Fuzzy Scale-free Graph, *Technical Report No. MIU/TR-03/08*, Machine Intelligence Unit, Indian Statistical Institute, Kolkata, India.

[Baskerville, 2005] Baskerville,S. and Bartel,D.P. (2005) Microarray profiling of microRNAs reveals frequent coexpression with neighboring miRNAs and host genes, *RNA*, **11**, 241-247.

[Brock *et al.*, 2008] Brock,G.N. *et al.* (2008) Which missing value imputation method to use in expression profiles: a comparative study and two selection schemes, *BMC Bioinformatics*, **9**.

[Datta *et al.*, 2006] Datta,P. and Datta,S. (2006) Evaluation of clustering algorithms for gene expression data, *BMC Bioinformatics*, **7**, S17.

[Eisen *et al.*, 1998] Eisen,M.B. *et al.* (1998) Cluster analysis and display of genome-wide expression patterns, *Proc. Natl Acad. Sci. USA*, **95**, 14863-14868.

[Handl *et al.*, 2005] Handl,J. *et al.* (2005) Computational cluster validation in post-genomic data analysis, *Bioinformatics*, **21**, 3201-3212.

[Karolchik *et al.*, 2004] Karolchik,D. *et al.* (2004) The UCSC Table Browser data retrieval tool, *Nucleic Acids Res.*, **32**, D493-D496.

[Laurent *et al.*, 2008] Laurent,L.C. *et al.* (2008) Comprehensive MicroRNA Profiling Reveals a Unique Human Embryonic Stem Cell Signature Dominated by a Single Seed Sequence, *Stem Cells*, **26**, 1506-1516.

[McGill *et al.*, 1978] McGill,R. *et al.* (1978) Variations of Boxplots, *The American Statistician*, **32**, 12-16.

[Perkins *et al.*, 2007] Perkins,D.O. *et al.* (2007) microRNA expression in the prefrontal cortex of individuals with schizophrenia and schizoaffective disorder, *Genome Biol.*, **8**, R27.

[Rousseeuw, 1987] Rousseeuw,P. (1987) Silhouettes: a graphical aid to the interpretation and validation of cluster analysis, *J. Comput. Appl. Math.*, **20**, 53-65.

[Shalgi *et al.*, 2007] Shalgi,R. *et al.* (2007) Global and Local Architecture of the Mammalian microRNA-Transcription Factor regulatory Network, *PLoS Comput. Biol.*, **3**, e131.

[Troyanskaya *et al.*, 2001] Troyanskaya,O. *et al.* (2001) Missing value estimation methods for DNA microarrays, *Bioinformatics*, **17**, 520-525.
